# Supplementary material for: Designing highly multiplex PCR primer sets with Simulated Annealing Design using Dimer Likelihood Estimation (SADDLE)
Source: Nat Commun. 2022 Apr 11;13:1881. doi: 10.1038/s41467-022-29500-4 (PMC9001684; doi:10.1038/s41467-022-29500-4)

Designing Highly Multiplex PCR Primer Sets with Simulated Annealing Design using Dimer Likelihood Estimation (SADDLE)

Nina G. Xie*,[1] Michael X. Wang*,[1] Ping Song,[1] Shiqi Mao,[2] Yifan Wang,[3]
Yuxia Yang,[3] Junfeng Luo,[3] Shengxiang Ren+,[2] and David Yu Zhang+[1,4]

[1]Department of Bioengineering, Rice University, Houston, TX
[2]Department of Medical Oncology, Shanghai Pulmonary Hospital, Shanghai, China
[3]NuProbe China, Shanghai, China
[4]NuProbe USA, Houston, TX
** these authors contributed equally to this work*
+ correspondence and requests for materials should be addressed to SR (email: harry_ren@126.com) and DYZ (email: dyz1@rice.edu)

(Dated: March 8, 2022)

S1. Experiment Protocols 1
S2. Data Analysis Workflow 3
S3. Analysis of 96 plex On-Target amplicons, Dimers and Non-specfic amplicons 5
S4. Results of libraries with adapter PCR-based protocol 11
S5. Clinical FFPE samples libraries prepared with PS3 14
S6. Results of 384-plex primer set NGS experiments 16
S7. Badness evaluation of PS2 and PS3 19
S8. Gene fusions detected and confirmed by Sanger Sequencing 20
S9. Primer candidates dG Calculation 24
S10. AmpliSeq Comparison Experiments 26
S11. Gene fusions Sanger Traces 31

S1. Experiment Protocols

In this paper, we performed validation of SADDLE optimized primer sets on NGS with 2 types of library preparation protocols to add adapters: ligation-based and PCR-based.

Library preparation

Sample preparation. Genomic DNA used in NGS experiments was NA18562 (Coriell Biorepository). All Genomic DNA was sheared using the Covaris M220 Focused-Ultrasonicator and Holder XTU Insert microTUBE 130L to achieve the product with the length peak at 150 nt, and quantified using Qubit 3.0 Fluorometer (Thermo Fisher Scientific) and Qubit dsDNA BR Assay Kit (Thermo Fisher Scientific). Sheared genomic DNA was used as template input in both ligation-based and PCR-based library preparation protocol.

Ligation-based library preparation. As showed in Supplementary Fig. S1, sheared genomic DNA was used for template input of first multiplex PCR. Multiplex PCR products were purified using DNA Clean & Concentrator-5 column (Zymo Research). Downstream end-prep ligation used NEBNext Ultra II DNA Library Prep Kit for Illumina, followed with column purification. The end product was quantified using qPCR and then amplified by index primers. Index PCR product was purified using AMPure XP (Beckman Coulter Life Sciences), and finally quantified using Qubit dsDNA HS Assay Kit.

PCR-based library preparation. As showed in Supplementary Fig. S1, after the multiplex PCR and column purification same as the ligation-based protocol, second PCR was performed to add adapters, followed with column purification. In our project, the PCR cycles and PCR procedures may vary depends on different DNA polymerases selection and primer concentration. The product was quantified using qPCR and then sufficiently amplified with index primers. PCR amplicons were purified using AMPure XP, and finally quantified using Qubit dsDNA HS Assay Kit.

In both NGS library preparation protocols, we used PowerUp SYBR Green Master Mix (Thermo Fisher Scientific) for all qPCR quantitations; Phusion Hot Start Flex DNA Polymerase (New England Biolabs), Vent(exo-) DNA Polymerase (New England Biolabs) with standard protocol for PCR amplifications, DEPC water as dilution/elution buffer for all PCR mixture, column or AMPure XP for purification.

Comparison of two protocols. In the second step to add adapters, we compared the influence of 2 protocols: ligation and PCR. Ligation allows the enrichment of amplicons and dimers regardless of their directions; while PCR requires the amplicons to have one fP binding site and one rP binding site on each side. The reason is that when we ordered primers, fPs were attached with adapter 1 and rPs were attached with adapter 2. As the identification of amplicons in Illumina Miseq requires the attachment of p5 and p7 on each side, amplicons amplified by 2 fP or 2 rP in the multiplex PCR reaction won't be observed in final output NGS data. As shown in Supplementary Fig. S2, species 2 and species 3 won't be observed in the FASTQ file when using PCR-based protocol. Utilizing PCR to add adapters will cause the missing of several species of dimers or non-specific amplicons, therefore it's not an ideal option to analyze dimers in this project.

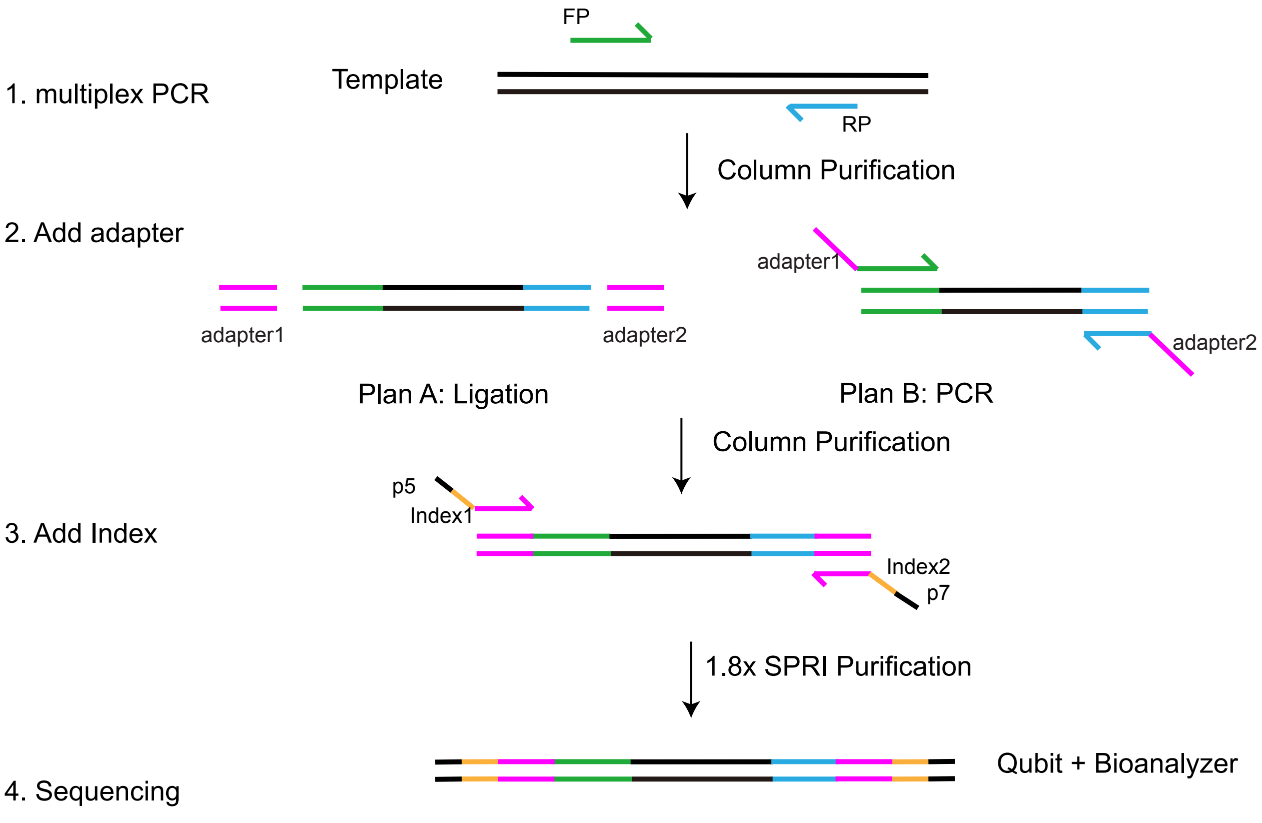

Supplementary FIG. S1. NGS library preparation protocol.

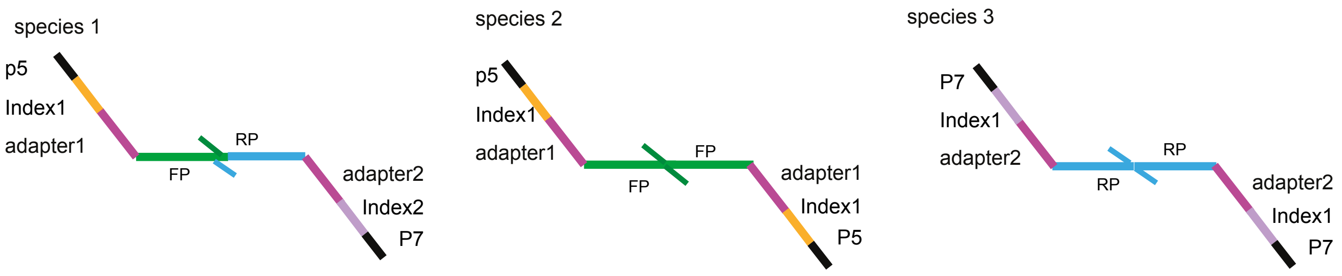

Supplementary FIG. S2: Potential dimer species in an NGS library

S2. Data Analysis Workflow

1. Dimer Candidates Generation. As showed in Supplementary Fig. S3, in our data analysis protocol, we did adapter trimming from raw FASTQ file and separated all the reads according to their length: we saved reads longer than 60 nt into long reads file, and reads shorter than 60 nt into short reads file. The choice of 60 nt reads length filter because of 2 reasons: 1), As in general primers are from 20 nt to 30 nt, therefore considering the overlap for at least 3 bp between two primers to dimerize, theoretically dimers are going to be shorter than 60 bp. 2), To increase the accuracy of Bowtie2 alignment[1]. We noticed that sometimes Bowtie2 can align the shorter dimer reads to the on-target reference inaccurately. As designed on-target amplicons were between 60 nt to 120 nt, therefore to prevent any potential inaccurate alignment, only the long reads files were aligned to reference sequences using Bowtie2.

2. Bowtie2 Alignment. Bowtie2 was used to do the paired-end alignment to the reference sequence. Unmapped long reads and short reads from previous steps together were termed as dimer candidates.

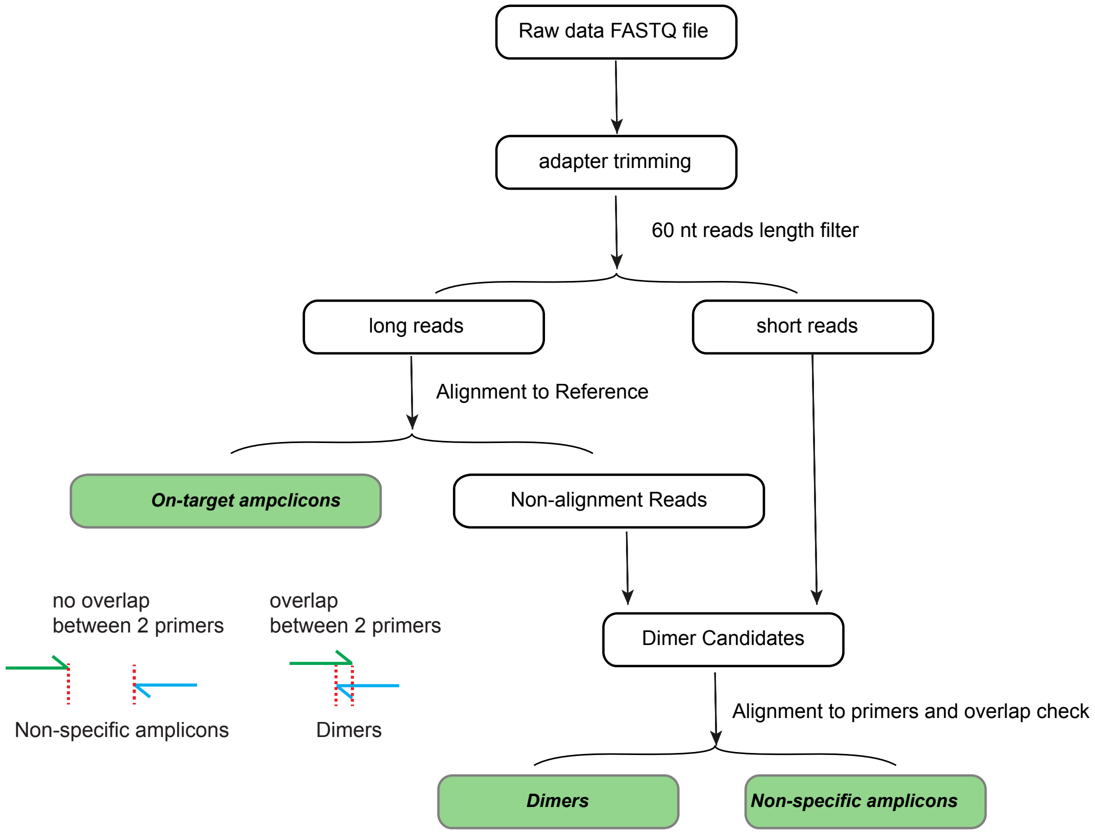

Supplementary FIG. S3: NGS data analysis workflow for Dimer Candidates generation

3. Categorization of dimers and non-specific amplicons. We showed in Supplementary Fig. S4 how we separated dimer candidates into dimers and non-specific amplicons. We first created a primer hash table, in which all the primers in a primer set are given a specific number according to their first 8 nt at 5' end. Therefore, this hash table saved all the first 8 nt information of all the primers. Noted that 8 nt at 5' end was an arbitrarily selected number. Considering the uniqueness of each primer, 8 nt was a relatively small value that we can identify each primer individually. If this number was too small, some primers shared the same first several bases couldn't be separated and would cause the collision; however, if this number was too large, the sequences would more likely to be involved in dimerization, as primer sequence approaching 3' end are more likely binding to each other.

After the creation of a hash table saving all the information of all the primers, we went over all the first 8 nt of paired-end dimer candidates reads and calculated them using the same function we created for the hash table. For an N-plex primer set, If both primers can be found in read 1 and read 2, either in the $2N \times 2N$ dimer matrix or the $2N \times 2N$ non-specific amplicon matrix, a particular position represented amplifications by two primers will add 1.

Importantly, once we successfully identified both primers in read 1 and read 2, we checked if there was any overlap between 2 primers. If an overlap more than 3 nt was between two primers, we identified this read to be a dimer, otherwise we identified it as a non-specific amplicon.

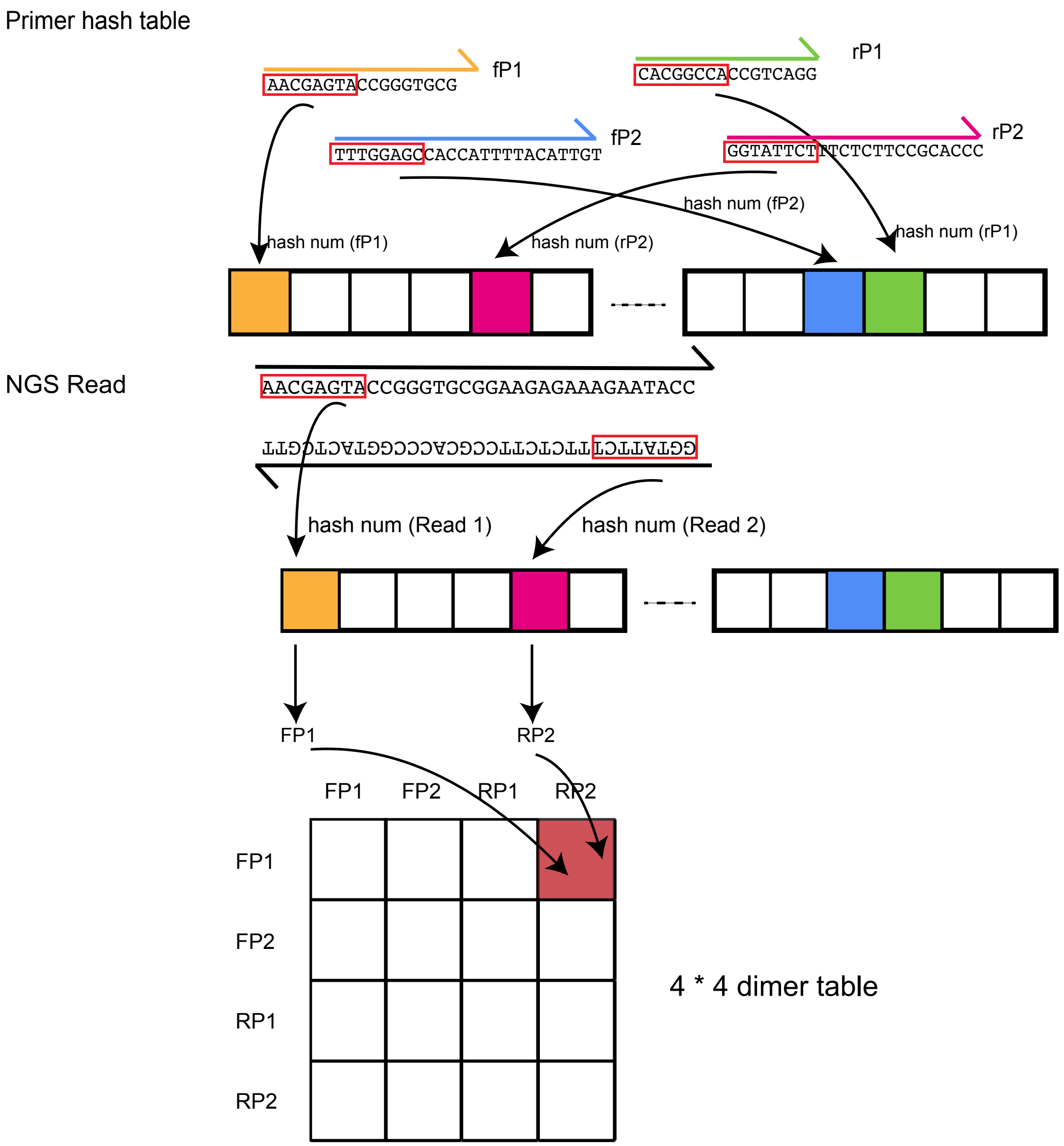

Supplementary FIG. S4: Categorization of dimers and non-specific amplicons

[1] Langmead, B., & Salzberg, S. L. (2012). Fast gapped-read alignment with Bowtie 2. Nature methods, 9(4), 357.

S3. Analysis of 96 plex On-Target amplicons, Dimers and Non-specific amplicons

Experimental Protocols.

1. Multiplex PCR. We prepared PCR reaction mix by combining forward primer mix, reverse primer mix, ThermoPol Reaction Buffer (10X) , Vent(exo-) DNA Polymerase (NEB), dNTP, template solution and Milli-Q H2O. The total reaction volume was 50 μL in a 0.2mL Eppendorf PCR tube as shown in Table S1. The PCR tubes containing the reaction mixtures were placed into T100 Thermocycler (Bio-Rad), amplified following the PCR protocol listed in Table S2. Multiplex PCR products were purified using DNA Clean & Concentrator-5 (ZYMO Research).

Reagent	Working Stock Concentration	Final Concentration	Add in Volume(μL)
ThermoPol Reaction Buffer	10x	1x	5
Deoxynucleotide (dNTP)	10 mM	0.2mM	1
Forward Primer Mix	100nM	30nM	15
Reverse Primer Mix	100nM	30nM	15
DNA Template	5ng/μL	0.2ng/μL	2
Vent(exo-) DNA Polymerase	-	1 unit	0.5
Milli-Q H2O	-	-	11.5
Total Volume			50

Supplementary TABLE S1: Multiplex PCR mixture formulation

Step	Temperature	Duration
1. Initial Denaturation	95°C	3 min
2. Denaturation	95°C	30 s
3. Annealing	60°C	3 min
4. Extension	72°C	30 s
Repeat Steps 2 to 4 for 17 times		
8. Final Extension	72°C	5 min
Hold at 4°C		

Supplementary TABLE S2: Multiplex PCR reaction protocol

2. End-Repair and Ligation. We prepared the reaction mixture by combining NEBNext Ultra II End Prep Enzyme Mix, NEBNext Ultra II End Prep Reaction Buffer, purified multiplex PCR products and Milli-Q H2O. The total reaction volume was 60 μL in a 0.7mL Eppendorf PCR tube as shown in Table S3. The PCR tubes containing the reaction mixtures were placed into Personal Eppendorf MasterCycler Thermocycler, and the thermocycling started at 20°C for 30 min and 65 °C for 30 min, with the heated lid set to 80 °C. The reactions were then hold at 4°C.

Reagent	Add in Volume(μL)
NEBNext Ultra II End Prep Enzyme Mix	3
NEBNext Ultra II End Prep Reaction Buffer	7
Purified PCR Products	20
Milli-Q H2O	30
Total Volume	60

Supplementary TABLE S3: End-Repair mixture formulation

End repair mixture was ligated with adapters using NEBNext® Ultra™ II Ligation Module (NEB). Each reaction was a mixture of previous end repair mixture, NEBNext Ultra II Ligation Master Mix, NEBNext Ligation Enhancer and NEBNext Adaptor for Illumina as shown in Table S5. Ligation was performed on a personal Eppendorf MasterCycler Thermocycler. Thermocycling started with the incubation at 20°C for 15 min with the heated lid off; after adding 3μl USER™ enzyme to the ligation mixture, the reactions were incubated at 37°C for

Reagent	Add in Volume(μL)
End Prep Reaction Mixture	60
NEBNext Ultra II Ligation Master Mix	30
NEBNext Ligation Enhancer	1
NEBNext Adaptor for Illumina	2.5
Total Volume	93.5

Supplementary TABLE S4: Ligation mixture formulation

15 min with the heated lid set to 55°C. Ligation products were purified using DNA Clean & Concentrator Kits-5 (ZYMO Research).

3. Index PCR. Index PCR was performed to sufficiently enrich the ligated products. Each reaction was a mixture of p5 and p7 index primers using NEBNext® Multiplex Oligos for Illumina® (NEB), purified ligation products, PCR reagents including Vent(exo-) DNA polymerase, ThermoPol Reaction Buffer, dNTPs and Milli-Q H2O. The total volume of each reaction was 52μL in a 0.2mL Eppendorf PCR tube as shown in Table S5. Index PCR was conducted on a T100 Thermocycler or a C1000 Thermocycler (Bio-Rad), followed the protocol in Table S6. Noted that the N in Table S6 varied depend on the previous qPCR Ct value for quantitation of the ligated products. Index PCR products were purified using AMPure XP beads (Beckman Coulter). 1.8X beads were added to the Index PCR reaction mixture, 40μL Milli-Q water was used individually as elution buffer for each library.

Reagent	Add in Volume(μL)
ThermoPol Reaction Buffer(10x)	5
dNTP(10mM)	1
i5 Index primer	2
i7 Index primer	2
DNA Template	5
Vent(exo-) DNA Polymerase	0.5
Milli-Q H2O	36.5
Total Volume	52

Supplementary TABLE S5: Index PCR mixture formulation

Step	Temperature	Duration
1. Initial Denaturation	95°C	3 min
2. Denaturation	95°C	30 s
3. Annealing	60°C	30 s
4. Extension	72°C	30 s
Repeat Steps 2 to 4 for N times		
8. Final Extension	72°C	5 min
Hold at 4°C		

Supplementary TABLE S6: Index PCR reaction protocol

4. Library quantitation and sizing measurement. All the libraries were quantified using the Qubit™ dsDNA HS Assay Kit (Thermo Fisher Scientific). Sizes of NGS libraries were measured using Bioanalyzer High Sensitivity DNA Assay (Agilent). DNA chip was run on the Agilent 2100 Bioanalyzer system. Products sizing of the PS1, PS2 and PS3 prepared libraries are shown in Supplementary Fig. S5.

5. Next generation Sequencing. All the libraries were loaded on a Miseq Reagent V2 for obtaining pair-end reads and were sequenced on a Miseq (Illumina).

Analysis of 96 plex On-Target amplicons, Dimers and Non-specific amplicons.

Length distribution of reads in the 3 primer set libraries. The NGS reads length distributions of the three species in the PS1, PS2, and PS3 libraries were consistent with the Bioanalyzer results. In these 3 libraries, on-target amplicons ranged from 90-120 nt, as designed; dimers ranged from 22-64 nt, while non-specific amplicons had a broader distribution, ranging from 30-130 nt (Supplementary Fig. S6).

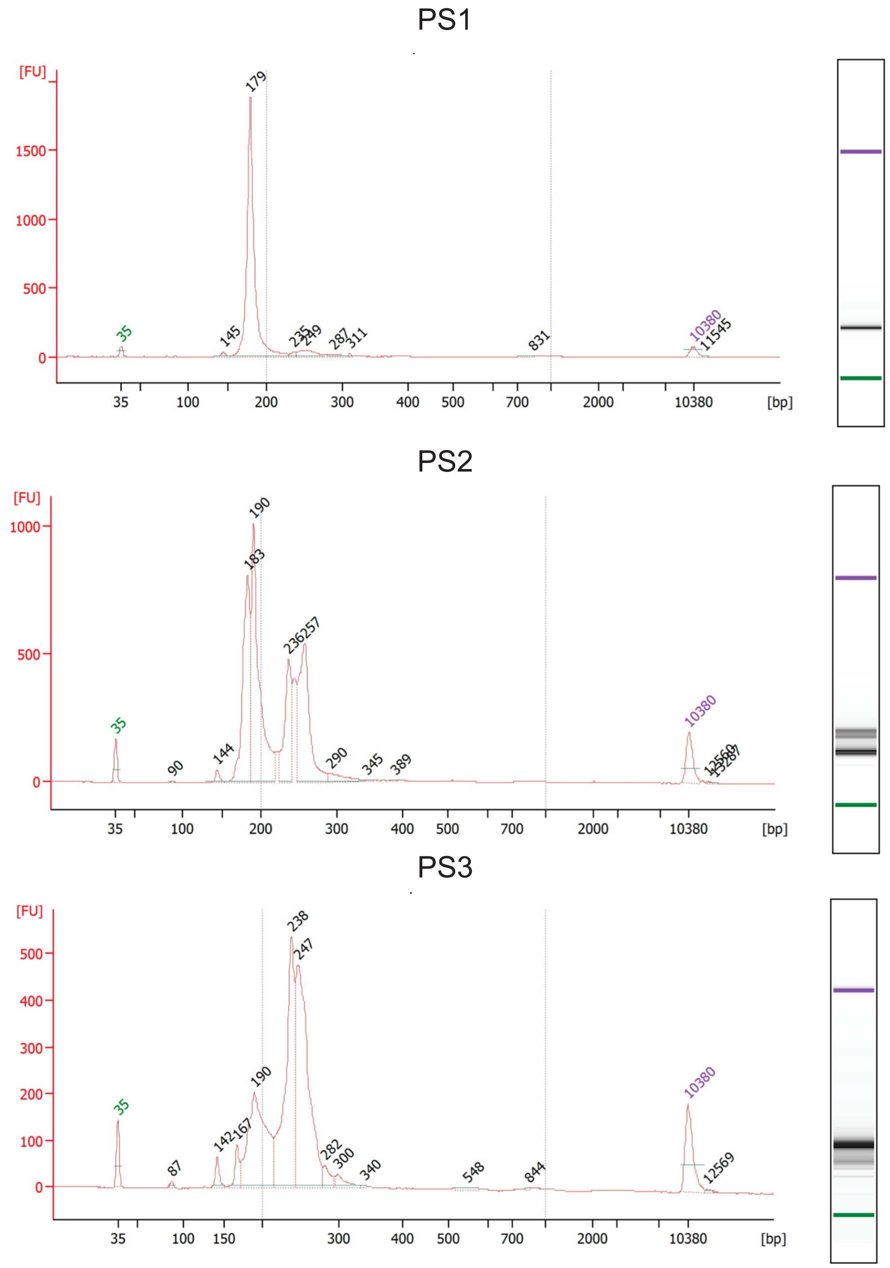

Supplementary FIG. S5: Bioanalyzer results of PS1, PS2 and PS3 after NGS library preparation

Uniformity of 96-plex on-target amplicons. As shown in Supplementary Fig. S7, amplicons were sorted according to their reads count. X-axis is the sorted 96 plex, y-axis is the log10 (Reads + 0.1). All the reads were normalized to reads count per one million total reads. PS3 library showed not only the highest on-target rate but also the best uniformity among each plex. Noted that after primer concentration modification, there were still 4 Off-target plex in PS1 library and 1 Off-target plex in PS2 library.

GC ratio of on-target amplicons. GC ratios of the amplicons were calculated in PS1, PS2 and PS3. In Supplementary Fig. S8, x-axis of the beeswarm plot is the 3 primer sets, y-axis is the GC contents. Because 3 primer sets were targeting approximately the same regions, there were no significant difference in amplicon's GC ratio among 3 primer sets.

Heatmap of non-specific amplicons. We showed in Supplementary Fig. S9 distributions of non-specific amplicons in PS1-PS3 libraries. As the most dominant species in PS1 library was dimer, non-specific amplicons in PS1 were much less than in PS2 and PS3. Non-specific amplicons in PS2 and PS3 were random and not comparable to each other. This is reasonable, as primers in PS2 and PS3 were different, and their combinations could potentially

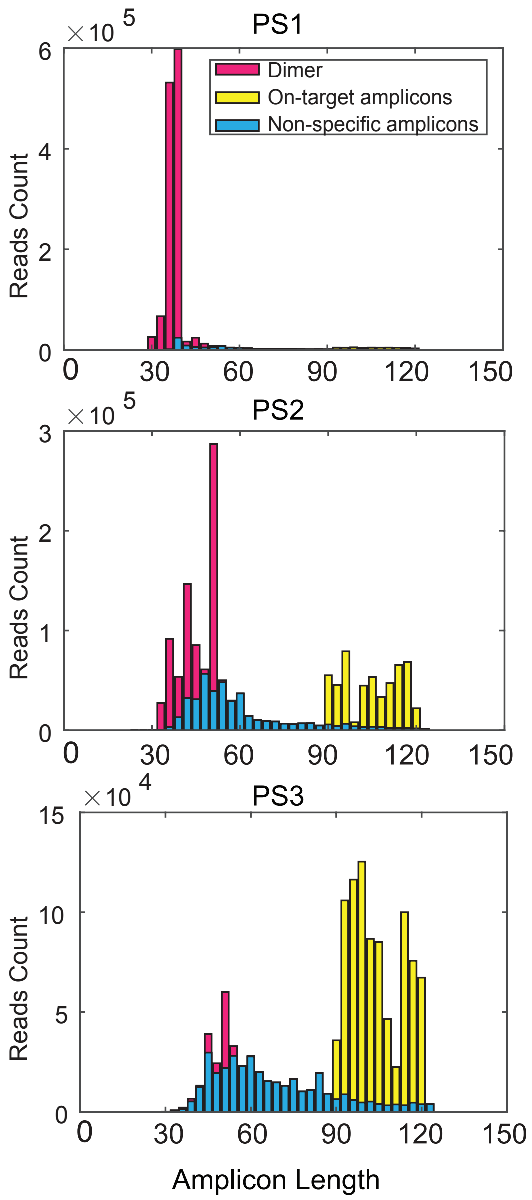

Supplementary FIG. S6: Length distribution of reads in the 3 primer set libraries

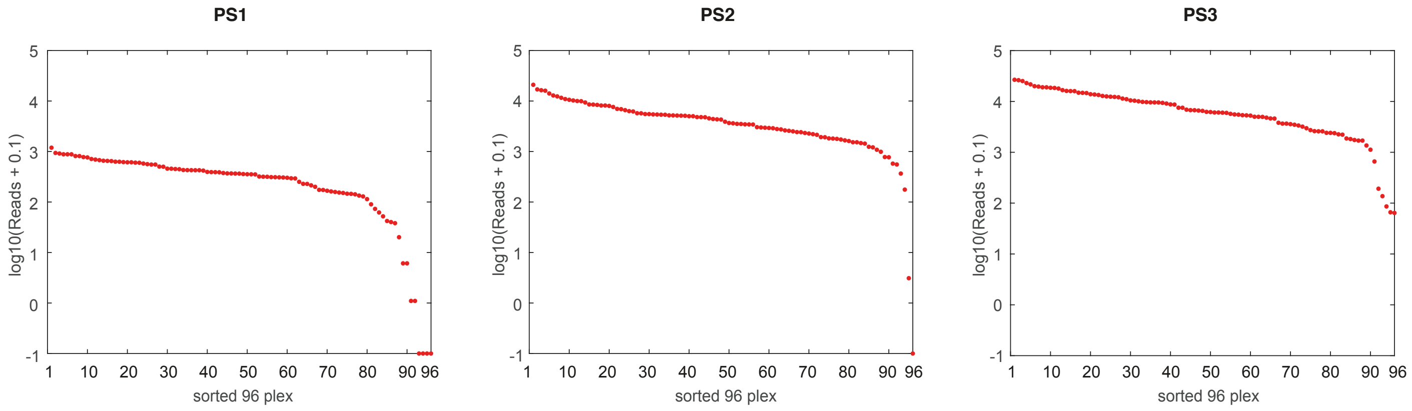

Supplementary FIG. S7: 96-plex On-target amplicons in 3 primer sets prepared NGS libraries

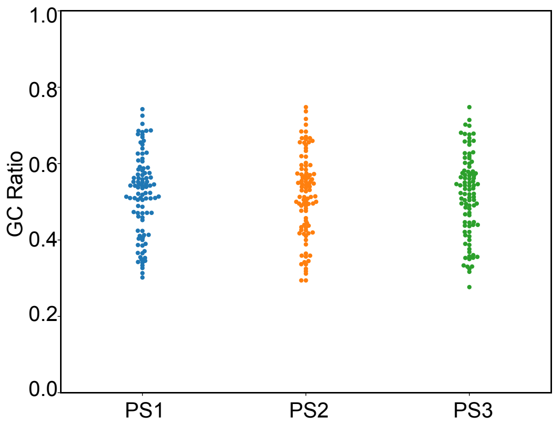

Supplementary FIG.S8: GC ratio of on-target amplicons in PS1, PS2 and PS3

amplify various kind of non-specific amplicons in genome. SADDLE doesn't include the elimination of non-specific amplicons, and we will try to do optimization of non-specific amplicons in the future.

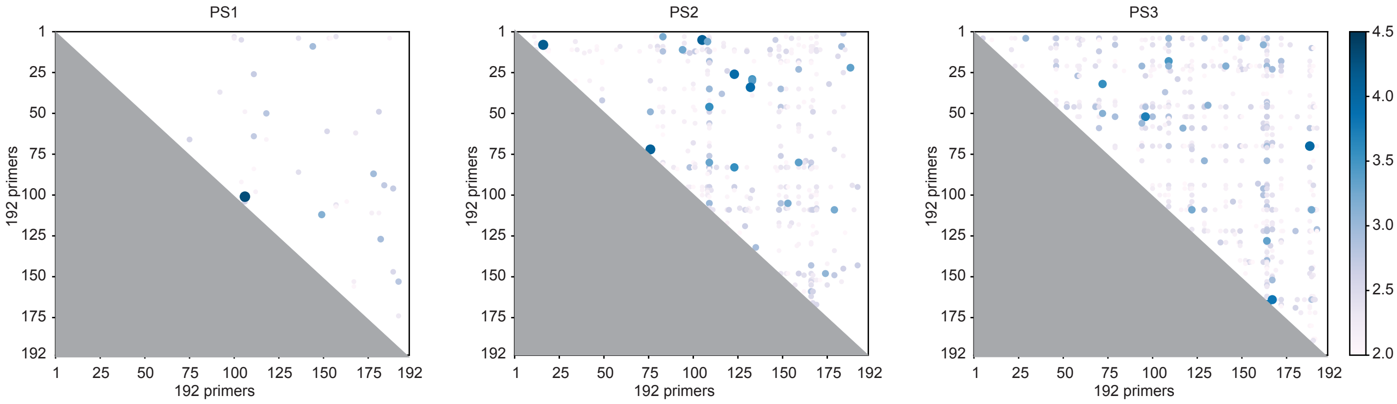

Supplementary FIG. S9: Heatmap of non-specific amplicons in 3 primer sets prepared NGS libraries

Heatmap of Dimers We showed in Supplementary Fig. S10 distributions of dimers in PS1-PS3 libraries. Dimer reads formed by the 192 $\times$ 192 primers in the 3 primer set libraries are shown in which primers 1-96 are fP 1-96 and primers 97-192 are rP 1-96. It is obvious that PS1 prepared library had the most dimers, while PS3 prepared libraries had the least dimers.

Distribution of reads observed in NGS library constructed using PS1, PS2, and PS3 (before primer concentration modification). Before primer concentration modification, the primer concentration was 30 nM in the PCR reaction. On Target reads are defined as those that aligned to the intended amplicons; Dimer reads are defined as those whose insert lengths are smaller than the sum of the two primer lengths; all other reads were classified as Non-specific. The vast majority of Non-specific reads align to other regions of the human genome, via a non-cognate pair of forward and reverse primers. The fraction of NGS reads mapped to Dimers dramatically decreases from PS1 to PS2 to PS3.

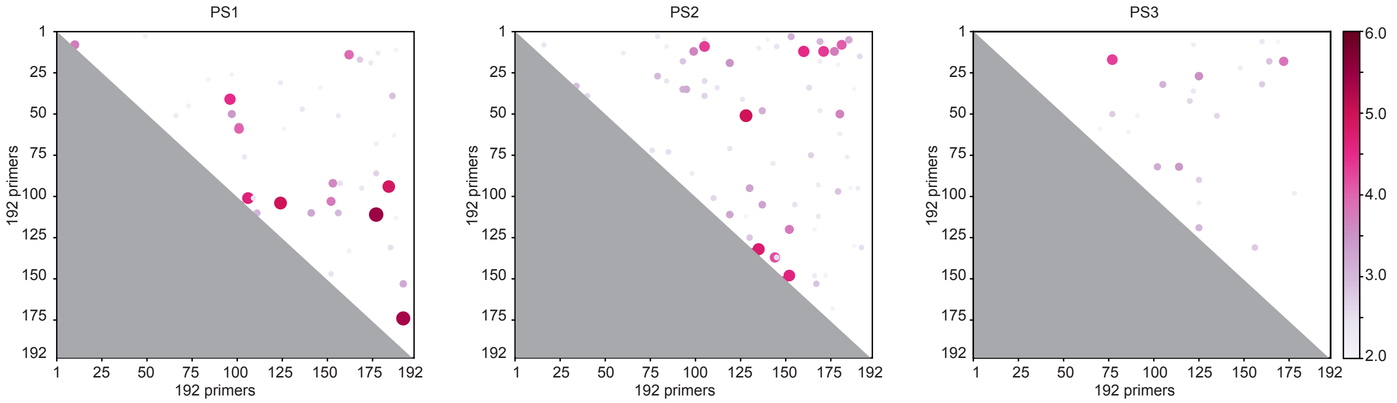

Supplementary FIG. S10: Heatmap of dimers in 3 primer sets prepared NGS libraries

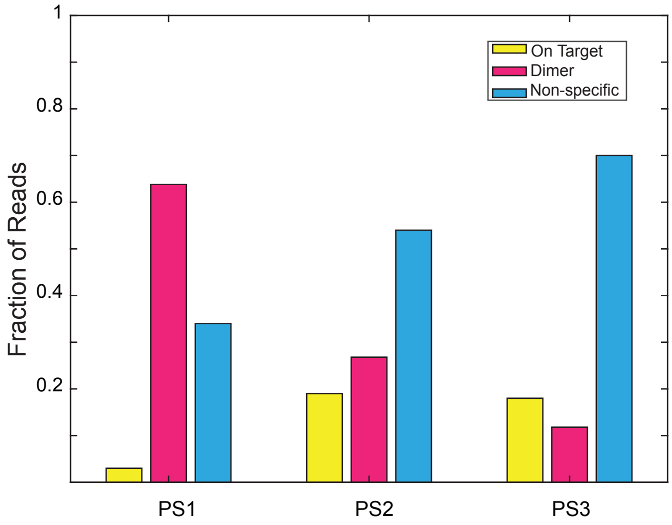

Supplementary FIG. S11: NGS-based characterization of amplicons generated from PS1, PS2, and PS3 prepared libraries before primer concentration modification. We separated NGS reads into 3 major species: on-target reads, dimer reads, and non-specific reads.

S4. Results of libraries with adapter PCR-based protocol

Experimental Protocols.

1. Multiplex PCR. We prepared PCR reaction mix by combining forward primer mix, reverse primer mix, ThermoPol Reaction Buffer (10X) , Vent(exo-) DNA Polymerase, dNTP, template solution and Milli-Q H2O. The total reaction volume was 50 μL in a 0.2mL Eppendorf PCR tube as shown in Table S7. The PCR tubes containing the reaction mixtures were placed into T100 Thermocycler, amplified following the PCR protocol listed in Table S8. Multiplex PCR products were purified using DNA Clean & Concentrator Kits-5.

Reagent	Working Stock Concentration	Final Concentration	Add in Volume(μL)
ThermoPol Reaction Buffer	10x	1x	5
Deoxynucleotide (dNTP)	10 mM	0.2mM	1
Forward Primer Mix	100nM	30nM	15
Reverse Primer Mix	100nM	30nM	15
DNA Template	8ng/μL	1.6ng/μL	10
Vent(exo-) DNA Polymerase	-	1 unit	0.5
Milli-Q H2O	-	-	3.5
Total Volume			50

Supplementary TABLE S7: Multiplex PCR mixture formulation

Step	Temperature	Duration
1. Initial Denaturation	95°C	3 min
2. Denaturation	95°C	30 s
3. Annealing	60°C	3 min
4. Extension	72°C	30 s
Repeat Steps 2 to 4 for 5 times		
8. Final Extension	72°C	5 min
Hold at 4°C		

Supplementary TABLE S8: Multiplex PCR reaction protocol

2. Index PCR. Index PCR was performed to enrich the purified Multiplex PCR products. Each reaction was a mixture of p5 and p7 index primers using Nextera XT Index Kit (illumina), purified multiplex PCR products, and PCR reagents including Vent(exo-) DNA polymerase, ThermoPol Reaction Buffer, dNTPs and Milli-Q H2O. The total volume of each reaction was 50μL in a 0.2mL Eppendorf PCR tube as shown in Table S9. PCR amplification was conducted on a T100 Thermocycler or a C1000 Thermocycler, followed the protocol in Table S10. Index PCR products were purified using 1.2X ratio of AMPure XP beads, 40μL Milli-Q water was used individually as elution buffer for each library.

Reagent	Add in Volume(μL)
ThermoPol Reaction Buffer(10x)	5
dNTP(10mM)	1
i5 Index primer	2
i7 Index primer	2
DNA Template	5
Vent(exo-) DNA Polymerase	0.5
Milli-Q H2O	34.5
Total Volume	50

Supplementary TABLE S9: Index PCR mixture formulation

Results. PCR-based library preparation to add adapters. We showed here the result of library using PCR to add adapters. In the heatmap of log10 (Reads count + 0.1) for all combinations of amplicons amplified

Step	Temperature	Duration
1. Initial Denaturation	95°C	3 min
2. Denaturation	95°C	30 s
3. Annealing	60°C	30 s
4. Extension	72°C	30 s
Repeat Steps 2 to 4 for 15 times		
8. Final Extension	72°C	5 min
Hold at 4°C		

Supplementary TABLE S10: Index PCR reaction protocol

by 192×192 primers in PS1, primers 1-96 are fP 1-96, primers 97-192 are rP 1-96. Each pixel is the read count number of amplicons that were amplified by two specific primers.

A zoomed-in plot of the first quadrant of the figure is on the left. Orange-yellow slash indicated the 96-plex on-target amplicons, amplified by the 96 fPs and their corresponding 96 rPs. Apart from 96-plex on-target amplicons, there were several color points indicated a number of dimers and non-specific amplicons in the NGS reads.

Importantly, as we used PCR to add adapters, only amplicons that were amplified by 1 fP and 1 rP can be seen in the heatmap. All the fP & fP or rP & rP dimers couldn't be observed in this figure. This situation was the same for non-specific amplicons: only if the non-specific amplicons were amplified by 1 fP and 1 rP could they be seen in final data. The missing species of dimers and non-specific amplicons suggested that PCR to add adapters was not an ideal method to observe dimers compared with ligation.

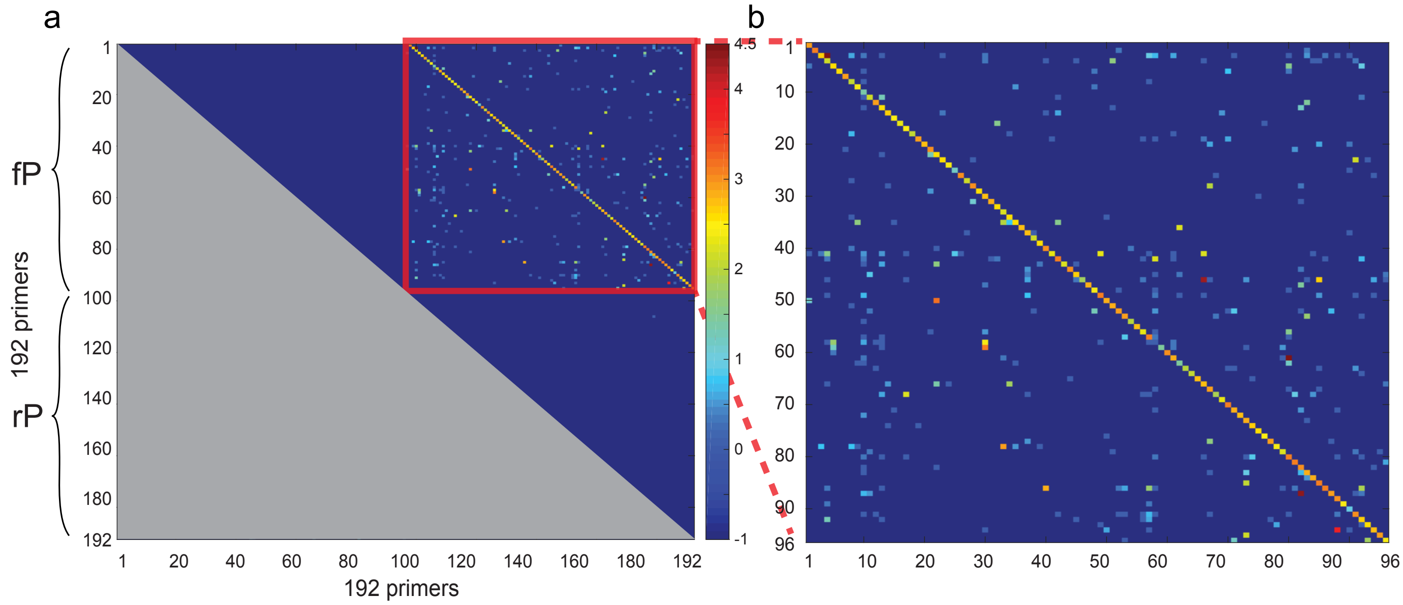

Supplementary FIG. S12: Heatmap of reads in PS1 library using PCR to add adapters

Dimers and non-specific amplicons distribution. We showed here heatmaps of dimers and non-specific amplicons in library using PCR to add adapters. In Supplementary Fig. S13, log10 (Reads count + 0.1) of dimer and non-specific amplicons amplified by 192×192 primers in PS1, primers 1-96 are fP 1-96, primers 97-192 are rP 1-96. Each pixel is the read count number of amplicons that were amplified by two specific primers. Noted that the colorbar are the same between 2 heatmaps, indicated the majority of the off-target reads were dimers, which is consistent with the results of library prepared with ligation-based protocol.

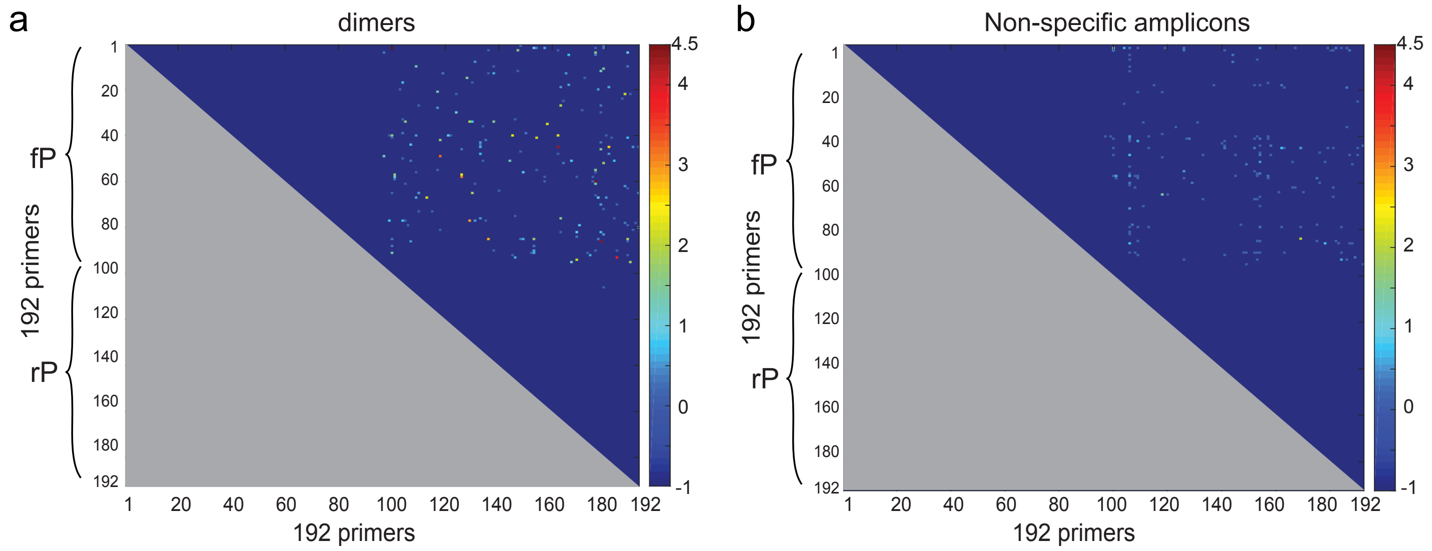

Supplementary FIG. S13: Heatmap of dimers and non-specific amplicons in PS1 library using PCR to add adapters

On-Target amplicons distribution. As shown in Supplementary Fig. S14, on-target amplicons were sorted in a descending order. On-target reads were normalized to be on-target reads per 1 million reads.

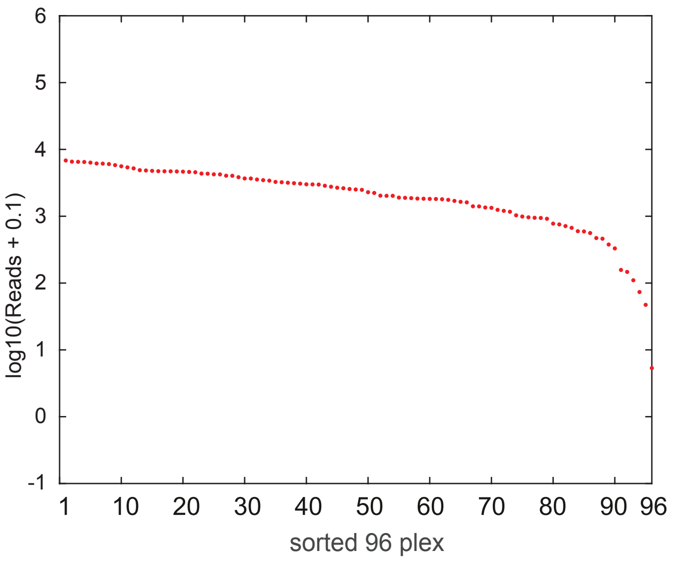

Supplementary FIG. S14: Sorted On-target amplicons in Adapter PCR-based library preparation protocol

S5. Clinical FFPE samples libraries prepared with PS3

Clinical FFPE samples libraries prepared with PS3.

We validated the optimized PS3 on 5 clinical samples (Origene). The cancer types of 5 samples included breast cancer, lung cancer and colorectal cancer, detailed information of sample types, patient age, patient gender and minimum stage grouping are shown in Table S11. FFPE blocks of each sample were extracted individually using GeneRead DNA FFPE Kit (Qiagen). The library preparation protocols for extracted DNA samples were the same as described in Section S3, with the input for individual reaction was 10 ng Qubit quantified DNA . Data analysis workflow was the same as described in Section S2.

We compared the on-target amplicons, dimers and non-specific amplicons in 5 NGS libraries. The highest on-target rate among the 5 clinical FFPE sample libraries was 85%, while the lowest was 29%. The variation of on-target rate may have been due to difference of FFPE samples' quality, because the FFPE samples have different amplifiable molecules even though we quantified each to be 10 ng by Qubit.

FFPE ID	Cancer type	Patient Age	Patient Gender	Minimum Stage Grouping
FFPE 1	Breast Cancer	54	Female	IIA
FFPE 2	Lung Cancer	57	Male	Not Reported
FFPE 3	Lung Cancer	75	Male	IB
FFPE 4	Colorectal Cancer	89	Female	IIA
FFPE 5	Colorectal Cancer	71	Male	IV

Supplementary TABLE S11: Summary of Clinical FFPE Samples.

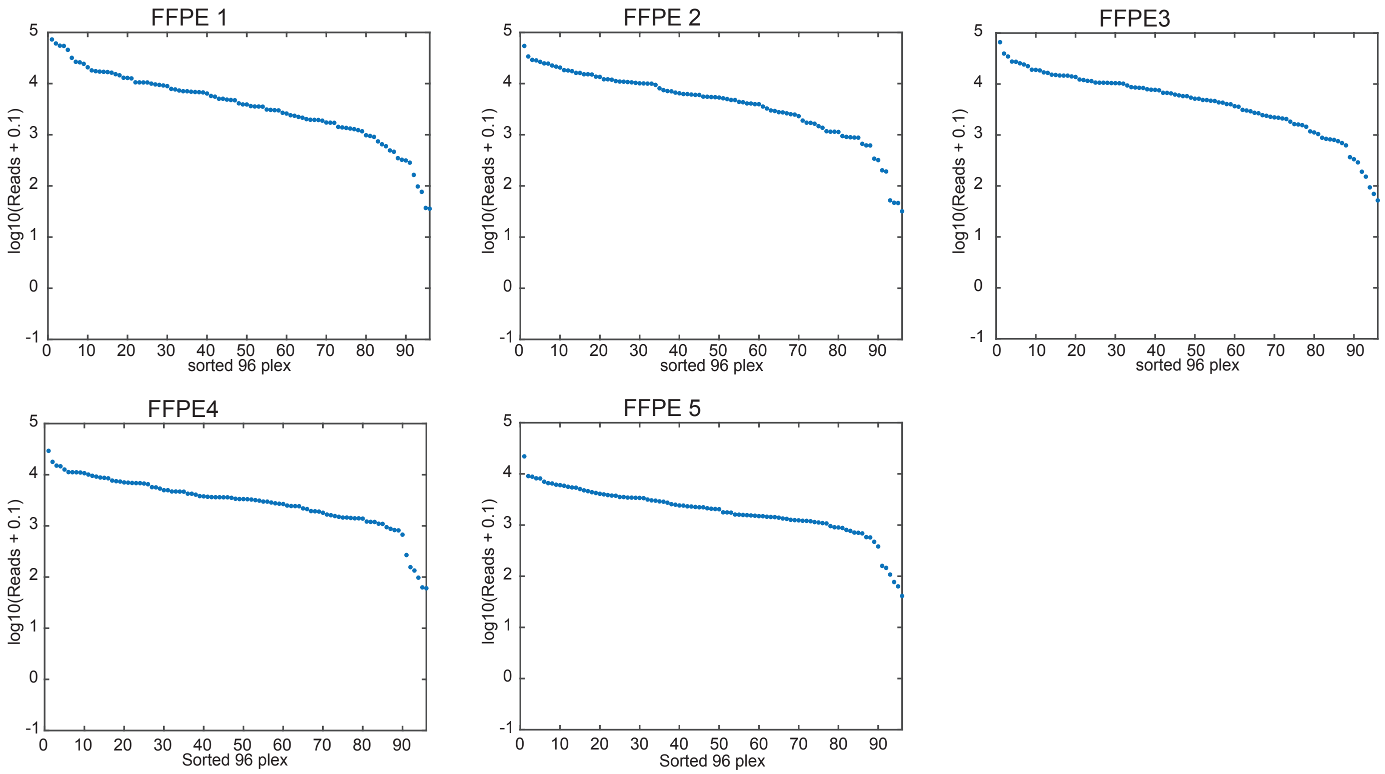

Supplementary FIG. S15: Sorted on-target amplicons of 5 FFPE sample libraries

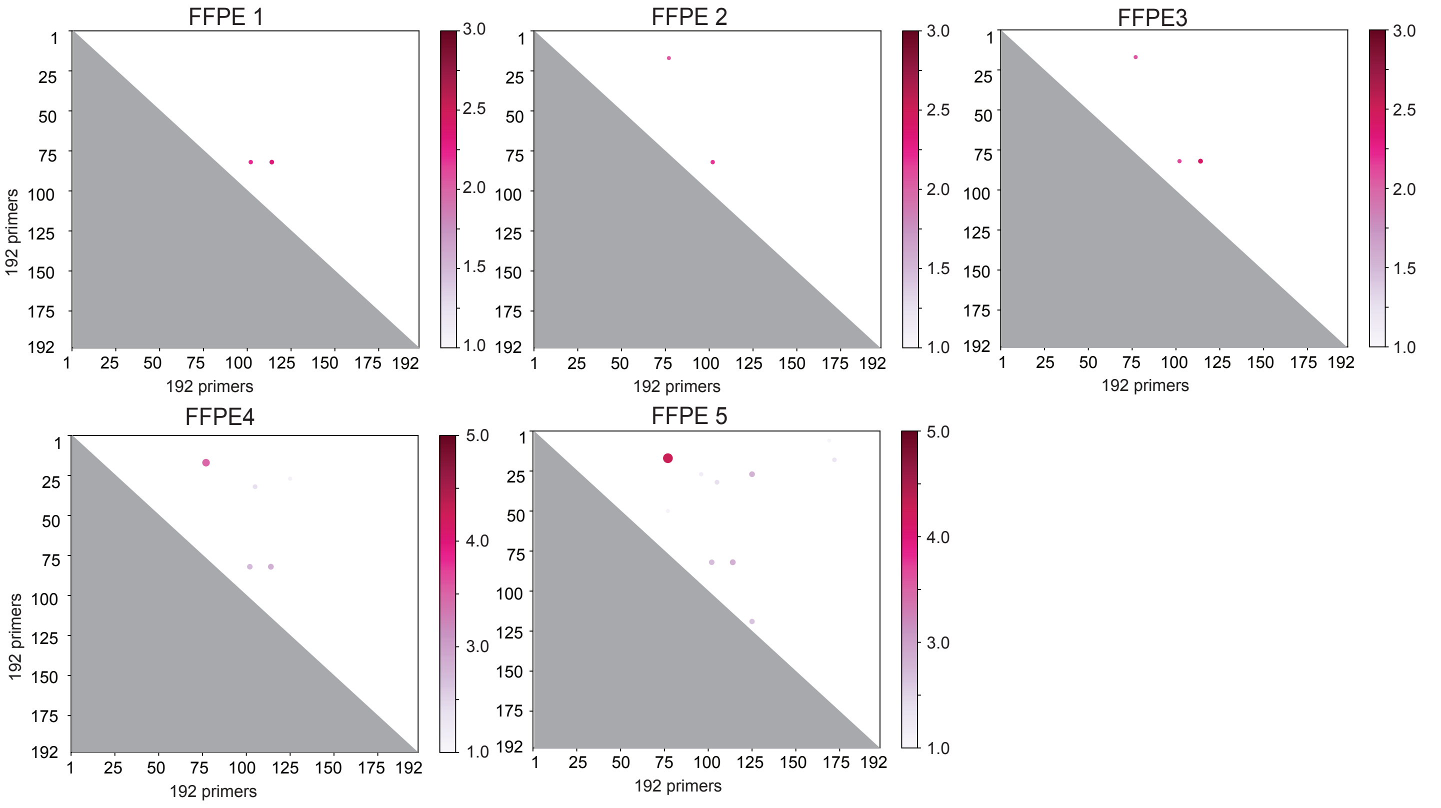

Supplementary FIG. S16: Dimers of 5 FFPE sample libraries

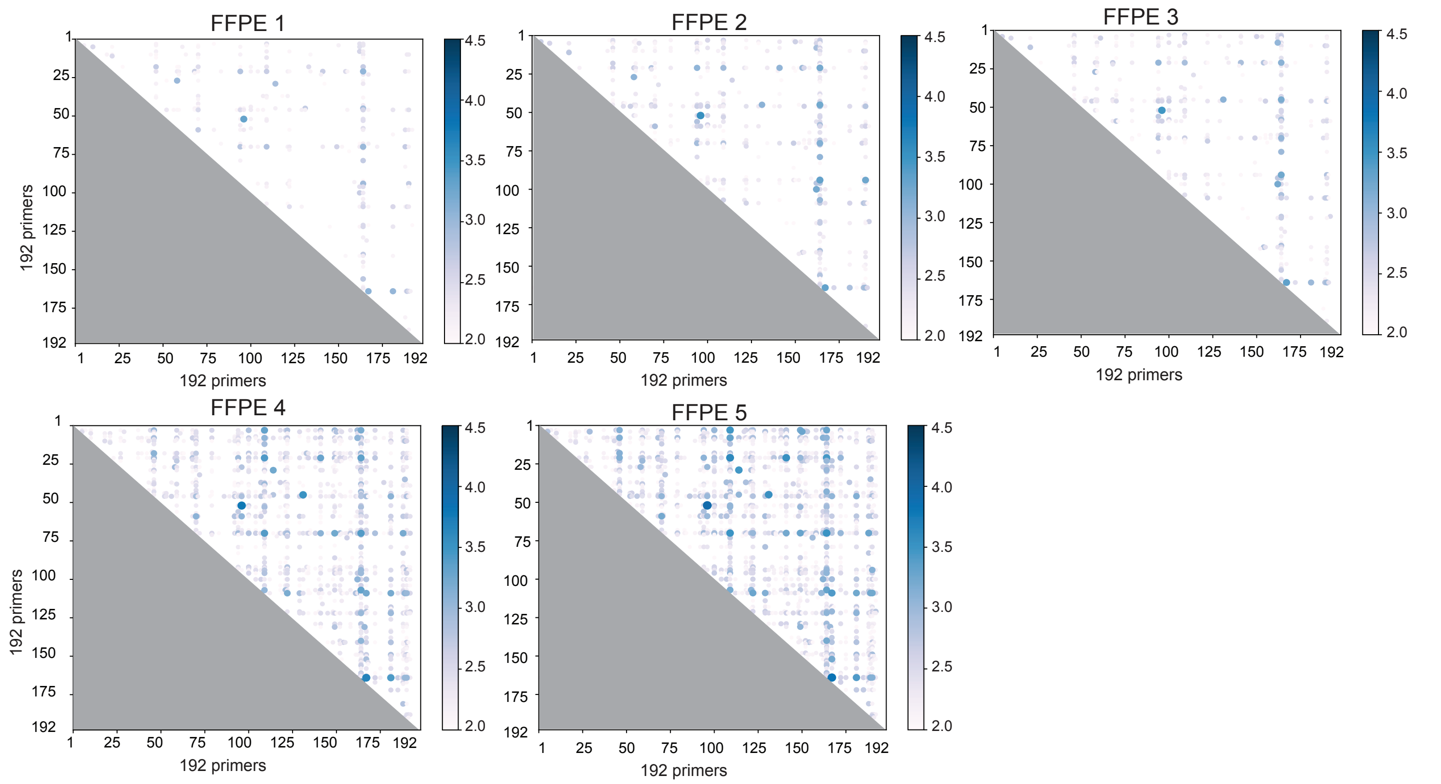

Supplementary FIG. S17: Non-specific amplicons of 5 FFPE sample libraries

S6. Results of 384-plex primer set NGS experiments

Experimental Protocols.

1. Multiplex PCR. We prepared PCR reaction mix by combining primer mix, Phusion buffer(5x), Phusion® Hot Start Flex DNA Polymerase (NEB), dNTP, template solution and Milli-Q H2O. The total reaction volume was 50 μL in a 0.2mL Eppendorf PCR tube as shown in Table S12. The PCR tubes containing the reaction mixtures were placed into T100 Thermocycler, amplified following the PCR protocol listed in Table S13. Multiplex PCR products were purified using DNA Clean & Concentrator Kits-5.

Reagent	Working Stock Concentration	Final Concentration	Add in Volume(μL)
Phusion HF Buffer	5x	1x	10
Deoxynucleotide (dNTP)	10 mM	0.2mM	1
Primer Mix	100nM	30nM	15
DNA Template	8ng/μL	0.8ng/μL	5
Phusion Hot Start Flex DNA Polymerase	-	1 unit	0.5
Milli-Q H2O	-	-	18.5
Total Volume			50

Supplementary TABLE S12: Multiplex PCR mixture formulation

Step	Temperature	Duration
1. Initial Denaturation	98°C	1 min
2. Denaturation	98°C	10 s
3. Annealing	60°C	3 min
4. Extension	72°C	30 s
Repeat Steps 2 to 4 for 20 times		
8. Final Extension	72°C	5 min
Hold at 4°C		

Supplementary TABLE S13: Multiplex PCR reaction protocol

2. End-Repair and Ligation. End-repair and ligation protocols for multiplex PCR products were the same as described in Section S3. Ligation product was purified using DNA Clean & Concentrator Kits-5.

3. Index PCR. Index PCR was performed to enrich the purified ligation products. Purified ligation product was diluted 100-fold. Each reaction was a mixture of p5 and p7 index primers using NEBNext® Multiplex Oligos for Illumina®, diluted purified ligation products, PCR reagents including Phusion buffer(5x), Phusion® Hot Start Flex DNA Polymerase, dNTPs and Milli-Q H2O. The total volume of each reaction was 52μL in a 0.2mL Eppendorf PCR tube as shown in Table S14. PCR amplification was conducted on a T100 Thermocycler or a C1000 Thermocycler, followed the protocol in Table S15. Index PCR reaction mixture was purified using 1.2X AMPure XP beads individually, 40μL Milli-Q water was used as elution buffer for each library.

Reagent	Add in Volume(μL)
Phusion HF Buffer (5x)	10
dNTP(10mM)	1
i5 Index primer	2
i7 Index primer	2
DNA Template	5
Phusion Hot Start Flex DNA Polymerase	0.5
Milli-Q H2O	31.5
Total Volume	52

Supplementary TABLE S14: Index PCR mixture formulation

4. Library quantitation and sizing measurement. All the libraries were quantified using the Qubit™ dsDNA HS Assay Kit. Sizes of NGS libraries were measured using Bioanalyzer High Sensitivity DNA Assay, and

Step	Temperature	Duration
1. Initial Denaturation	98°C	1 min
2. Denaturation	98°C	10 s
3. Annealing	60°C	30 s
4. Extension	72°C	30 s
Repeat Steps 2 to 4 for 8 times		
8. Final Extension	72°C	5 min
Hold at 4°C		

Supplementary TABLE S15: Index PCR reaction protocol

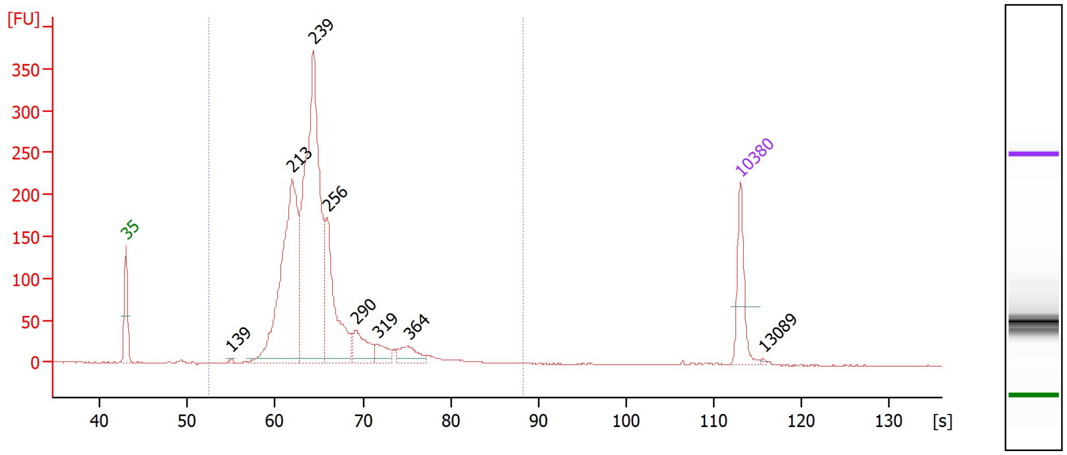

Supplementary FIG. S18: Bioanalyzer results of 384-plex primer set prepared library.

DNA chips were run on the Agilent 2100 Bioanalyzer system. Products sizing of the 384-plex primer set prepared library is shown in Supplementary Fig. S18.

Results.

Reads distribution in 384-plex primer set prepared libraries. We showed here the results of 384-plex primer set prepared NGS library. Within the total reads that passed QC and after trimmed adapter, the on-target rate of this library is 43%, the dimer rate is 1% and the non-specific amplicons rate is 56%. Heatmaps of dimers and non-specific amplicons amplified by 768 * 768 primers are shown in Supplementary Fig. S19, primers 1-384 were fP 1-384, primers 385-768 were rP 385-768. Each pixel is the read count number of amplicons that were amplified by two primers. Because ligation was used to add adapters in library preparation protocol, all the fP & fP and rP & rP species can be observed.

The length distribution of reads in this library is shown in Supplementary Fig. S20 on-target amplicon lengths were 60-120 nt as designed, and the dimer lengths were 26-67 nt, while the non-specific amplicon lengths were from 31-123 nt in three libraries.

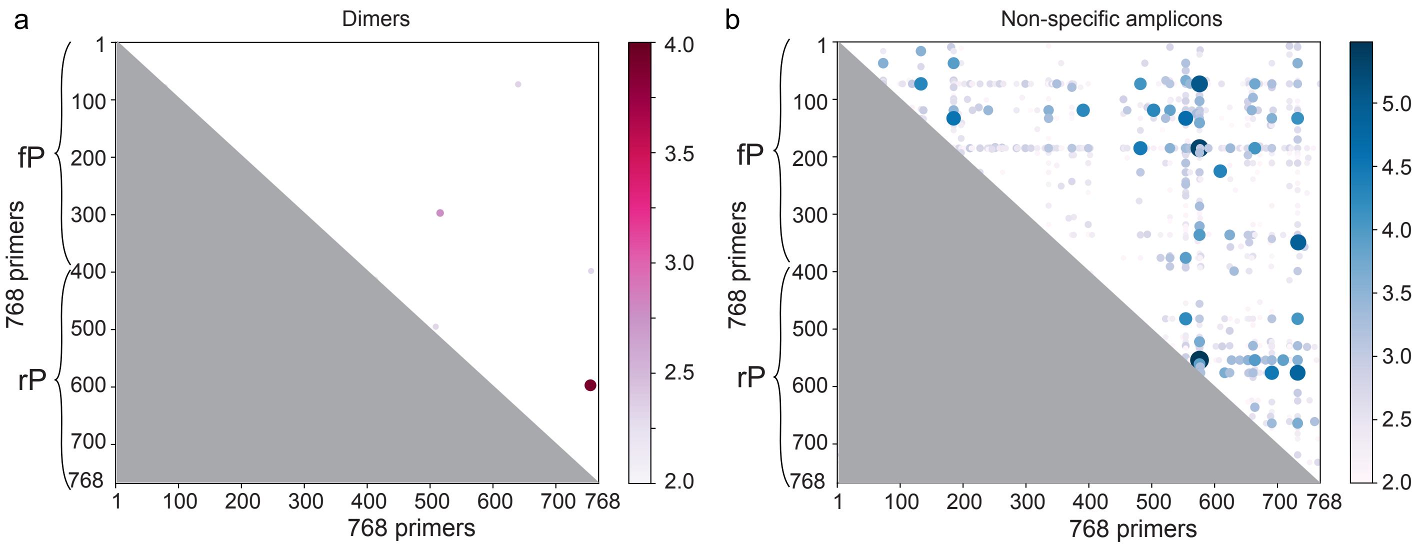

Supplementary FIG. S19: Heatmap of dimers and non-specific amplicons in 384-plex primer set prepared NGS libraries

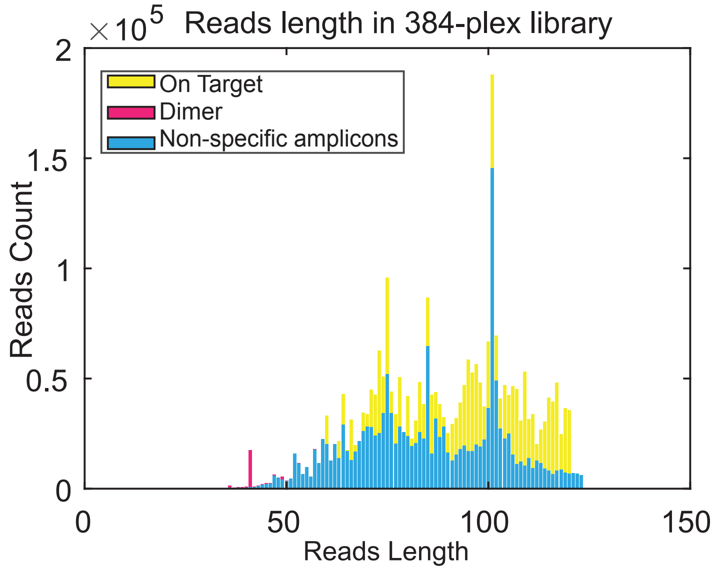

Supplementary FIG. S20: Length distribution of reads in the 384-plex primer set prepared NGS libraries.

S7. Badness evaluation of PS2 and PS3

Evaluation of Badness prediction in PS2 and PS3. For the best prediction of sensitivity and specificity of 36864 data points in PS2, true positive was 11, true negative was 35486, false positive was 1363 and false negative was 4. We plotted Receiver operator characteristic (ROC) accuracy of experimental dimers versus predicted badness in the determination of the dimer formation in the PS2, the AUC was 0.8146.

For the best prediction of sensitivity and specificity of 36864 data points in PS3, true positive was 4, true negative was 29477, false positive was 7377 and false negative was 6. We plotted ROC accuracy of experimental dimers versus predicted badness in the determination of the dimer formation in the PS3, the AUC was 0.5417.

The badness predictions of PS2 and PS3 were not ideal as when we optimize the dimer formation from PS1, we eliminated most possible formed dimers. However, there was still several experiment dimers that are unexpected and couldn't be predicted by the algorithm. We can look into the false positive dimers in PS2 and PS3 to improve our algorithm.

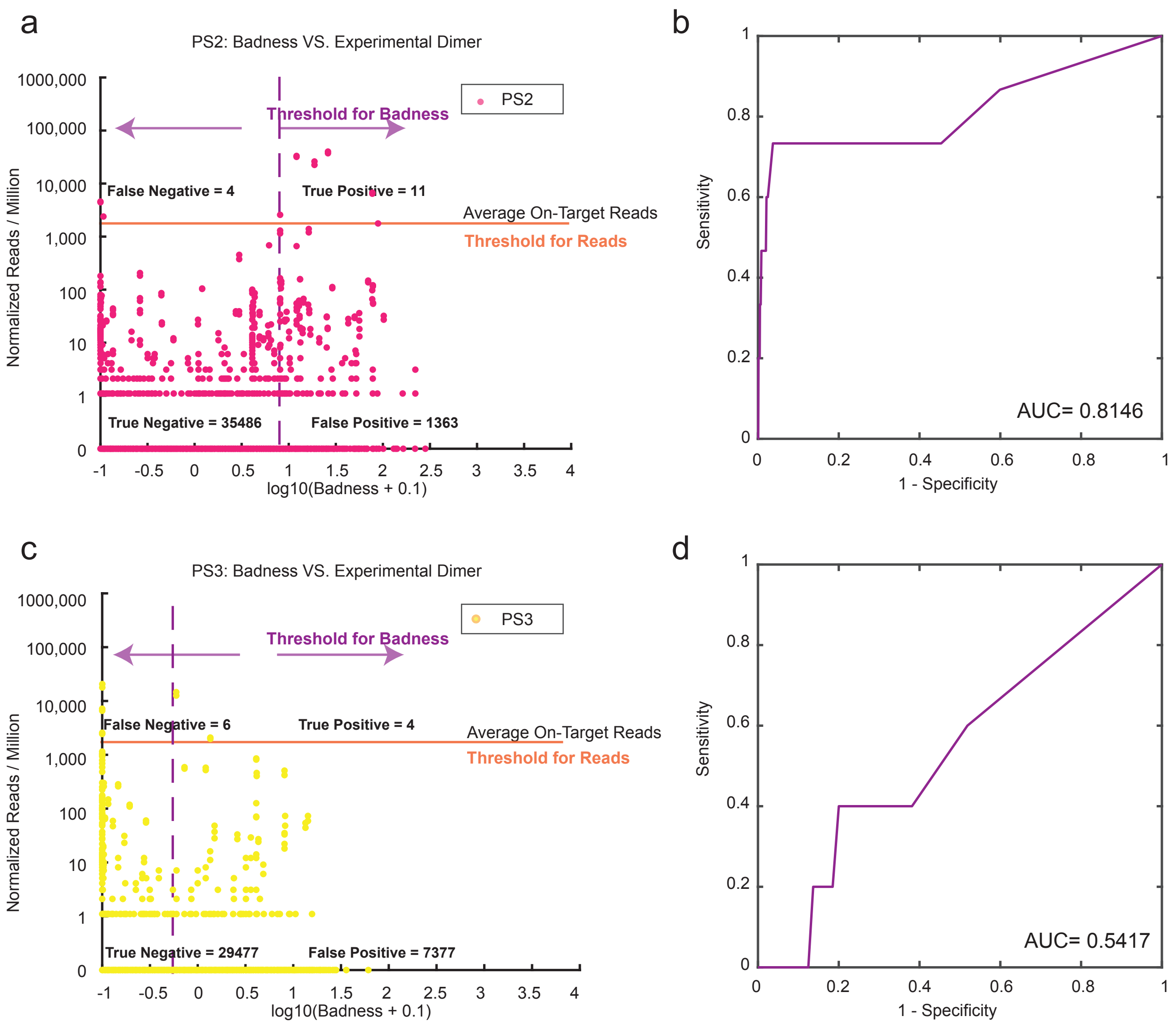

Supplementary FIG. S21: Evaluation of Badness prediction. **(a)** Comparison of experimental dimers and predicted Badness in PS2. All the counts of dimer reads were normalized to be dimer reads per 1000,000 reads. Orange line was a reference of the average on-target amplicons in this library, which was also set as the fixed Threshold for Reads (TR), purple line was the flexible Threshold for Badness (TB). **(b)** Receiver Operator Characteristic (ROC) accuracy of experimental dimers versus predicted Badness in the determination of dimerization in PS2. **(c)** Comparison of experimental dimers and predicted Badness in PS3.**(d)** ROC accuracy of experimental dimers versus predicted Badness in the determination of dimerization in PS3.

S8. Gene fusions detected and confirmed by Sanger Sequencing

List of Gene Fusions detected.

Fusion Variant ID	Fusion Type	Fusion Variant ID	Fusion Type
1	EML4 exon 13 – ALK exon 20(EML4-ALK Variant 1)	29	SDC4 exon 4 – ROS1 exon 34
2	EML4 exon 20 – ALK exon 20(EML4-ALK Variant 2)	30	SLC34A2 exon 4 – ROS1 exon 32
3	EML4 exon 6 – ALK exon 20(EML4-ALK Variant 3a)	31	SLC34A2 exon 4 – ROS1 exon 34
4	EML4 exon 6 – ALK exon 20(EML4-ALK Variant 3b)	32	SLC34A2 exon 13 del – ROS1 exon 32
5	EML4 exon 6 – ALK exon 20(EML4-ALK Variant 3c)	33	SLC34A2 exon 13 del – ROS1 exon 34
6	EML4 exon 15 – ALK exon 20(EML4-ALK Variant 4)	34	CD74 exon 6 – ROS1 exon 34
7	EML4 exon 18 – ALK exon 20(EML4-ALK Variant 5)	35	TPM3 exon 8 – ROS1 exon 35
8	EML4 exon 2 – ALK exon 20(EML4-ALK Variant 5a)	36	TPM3 exon 2 – ROS1 exon 36
9	EML4 exon 14 – ALK exon 20	37	GOPC exon 7 – ROS1 exon 35
10	KIF5B exon 15 – ALK exon 20	38	GOPC exon 4 – ROS1 exon 36
11	KIF5B exon 17 – ALK exon 20	39	EZR exon 10 – ROS1 exon 34
12	KIF5B exon 24 – ALK exon 20	40	LRIG3 exon 16 – ROS1 exon 35
13	TFG exon 4 – ALK exon 20	41	KIF5B exon 15 – RET exon 11
14	HIP1 exon 28 – ALK exon 20	42	KIF5B exon 15 – RET exon 12
15	KLC1 exon 9 – ALK exon 20	43	KIF5B exon 16 – RET exon 12
16	STRN exon 3 – ALK exon 20	44	KIF5B exon 22 – RET exon 12
17	ATIC exon 7 – ALK exon 20	45	KIF5B exon 23 – RET exon 12
18	CARS exon 17 – ALK exon 20	46	KIF5B exon 24 – RET exon 8
19	CLTC exon 31 – ALK exon 20	47	KIF5B exon 24 – RET exon 11
20	DCTN1 exon 26 – ALK exon 20	48	CCDC6 exon 1 – RET exon 12
21	MSN exon 11 – ALK exon 20	49	NCOA4 exon 6 – RET exon 12
22	NPM1 exon 5 – ALK exon 20	50	TRIM33 exon 16 – RET exon 12
23	TPM3 exon 7 – ALK exon 20	51	TPM3 exon 7 – NTRK1 exon 10
24	TPM4 exon 7 – ALK exon 20	52	LMNA exon 2 – NTRK1 exon 10
25	VCL exon 16 – ALK exon 20	53	NACC2 exon 4 – NTRK2 exon 13
26	SDC4 exon 2 – ROS1 exon 32	54	QKI exon 6 – NTRK2 exon 16
27	SDC4 exon 2 – ROS1 exon 34	55	ETV6 exon 4 – NTRK3 exon 15
28	SDC4 exon 4 – ROS1 exon 32	56	ETV6 exon 5 – NTRK3 exon 15

Supplementary TABLE S16: NSCLC-related gene fusions

Quantitation of 10% Fusion Variant samples. We define the Variant Allele Frequency (VAF) as fusion gene sequence / wildtype sequence, noted that wildtype sequence is the sequence of the 5' fused gene and its original adjacent exon sequence. The reason we used this sequence as wildtype sequence is that the expression level of fusions is more likely to be similar to its 5' fused gene, as they share the same promoter.

All the assumed 10% variant samples were the mixture of diluted gBlocks (Integrated DNA Technologies) and human cDNA were Random-primed qPCR Human Reference cDNA (Takara Bio). Each sample was quantitated separately. In Supplementary Fig. S22, we showed the workflow to quantitate VAF of a sample: 1 universal forward primer, 1 wildtype(WT) reverse primer and 1 fusion reverse primer were designed to amplify both the variant sequence and WT sequence simultaneously, as variant and WT share the same sequence of gene 1. Thus, for each variant sample, we used 3 primers with adaptor sequences attached targeting both Variant and WT simultaneously for library preparation. The efficiency of different primers can be neglected as the first PCR was only 2 cycles and the primer concentration were much higher than the template concentration. Index PCR and purification were conducted further for library preparation. Note that this quantitation process can be multiplexed.

As shown in Supplementary Fig. S22b, we used Unique molecular identifiers (UMI) attached in front of fP to quantitate variant reads and WT reads. We calculated Relative Quantity of gBlock as variant reads $\times$Plex Number/WT reads. Sample Variant Ratio was further calculated as $\frac{RelativeQuantity(gBlock)}{(RelativeQuantity(gBlock)+1)}$.

We presented the quantitation results of our presumed 10% Variant sample in Supplementary Fig. S22c. Noted that, our presumed value was according to our experience on gBlocks and genomic DNA, and it may be different with cDNA. As in genomic DNA, the copy number of different genes are in general the same, while the expression level of different genes vary, and this may lead to different copies of cDNA sequence [1].

The x axis is the sorted 56 plex, the y axis is the quantitated VAF. The real VAF range from 1% to 90%, which showed that the pre-quantitation was necessary as the expression level of different genes varies, thus the number of different genes in cDNA varies. After the quantitation of 10% VAF variant sample, we further did a serial dilution to 1% VAF sample and ready for use to test the design of our multiplex PCR-Sanger assay.

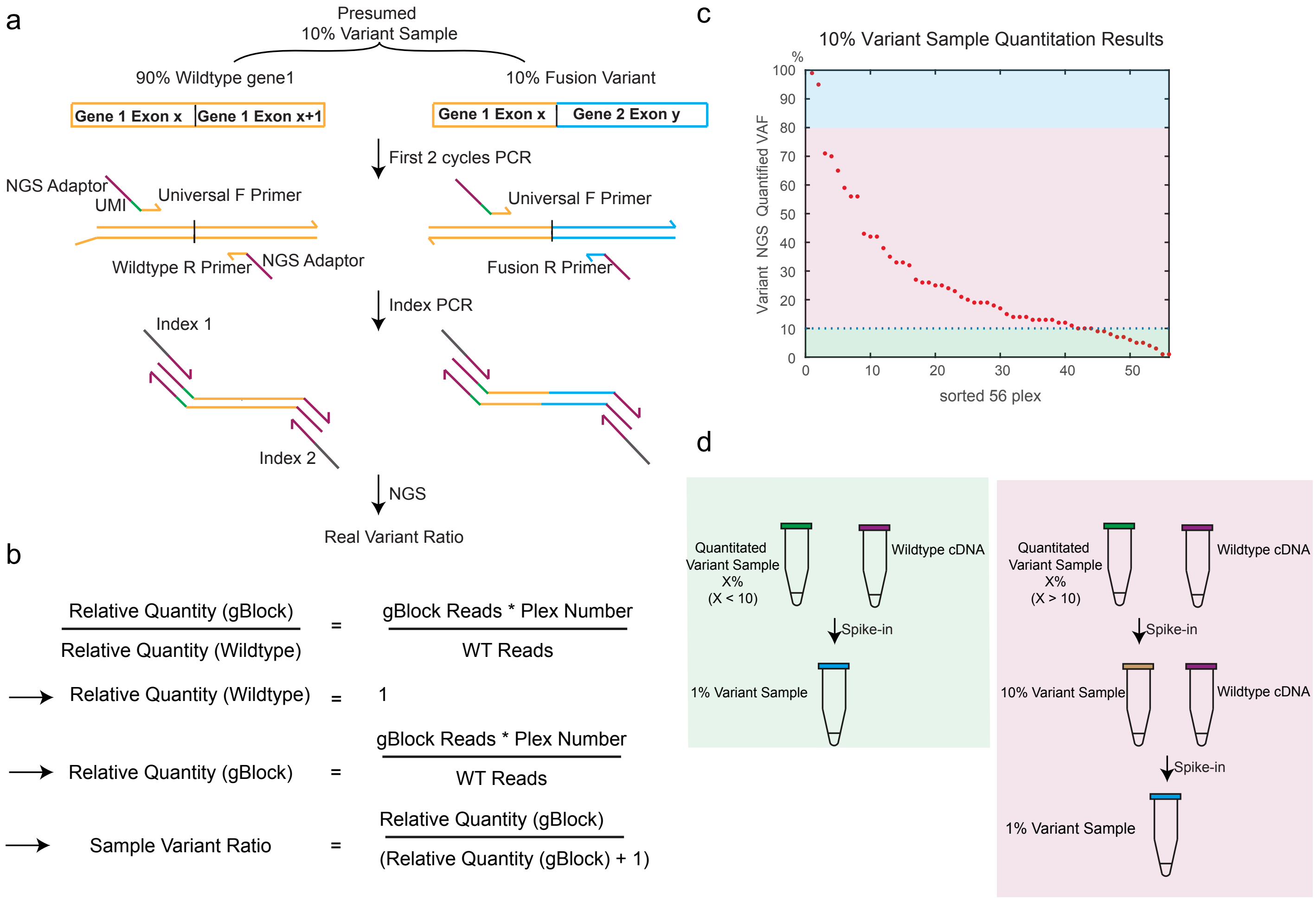

Supplementary FIG. S22: Quantitation of 10% Fusion Variant samples. **(a)** NGS workflow to quantitate synthetic fusion variant samples.**(b)** Calculation of synthetic sample's VAF. **(c)** NGS quantitation results of 56-plex presumed 10% fusion variants. **(d)** Further dilution to 1% VAF sample from NGS quantitation results.

Validation on Clinical samples. EV RNA was extracted separately from 10 NSCLC de-identified clinical plasma samples using exoRNeasy Serum/Plasma Kit (Qiagen). 10 EV RNA samples were reverse transcribed to cDNA using SuperScript™ IV Reverse Transcriptase (Thermo Fisher Scientific).

We prepared PCR reaction mix by combining multiplex primer mix, iTaq polymerase supermix , cDNA template solution and Milli-Q H2O.

The total reaction volume was 50μL in a 0.2mL Eppendorf PCR tube as shown in Table S17. Final mastermix were triplicated to be 15 μL/well to a hard-shell 96-well PCR plate (Bio-rad).

Reagent	Working Stock Concentration	Final Concentration	Add in Volume(μL)
2x iTaq polymerase supermix	2x	1x	25
Primer mix	250nM	25 nM	5
template	-	-	5
Milli-Q H2O	-	-	15
Total Volume	-	-	50

Supplementary TABLE S17: PCR reaction mixture formulation

The 96-well PCR plate containing the reaction mixtures was placed into T100 Thermocycler (Bio-Rad), amplified following the PCR protocol listed in Table S18.

Step	Temperature	Duration
1. Initial Denaturation	95°C	3 min
2. Denaturation	95°C	15 s
3. Annealing	60°C	1min
Repeat Steps 2 to 3 for 40 times		

Supplementary TABLE S18: PCR reaction mixture formulation

PCR product was purified using ExoSAP-IT™ PCR Product Cleanup Reagent (Thermo Fisher Scientific). 15 μL PCR products and 6 μL ExoSAP-IT were pooled together in a 0.2 mL Eppendorf PCR tube, and the PCR tubes containing the reaction mixtures were placed into T100 Thermocycler and incubated at 37°C for 4 min and 80°C for 1 min, then hold at 4°C.

Cycle sequencing was conducted on purified PCR products. A mixture of purified PCR products, 5 μM sequencing primer, BigDye™Terminator v1.1 Ready Reaction Mix and BigDye™ Terminator v1.1 5X Sequencing Buffer (Thermo Fisher Scientific) and Milli-Q H2O were pooled together in a 0.2 mL Eppendorf PCR tube. The total reaction volume was 10 μL as shown in Table S19. The amplification PCR protocol is shown in Table S20.

Reagent	Add in Volume(μL)
Purified products	1
Sequencing primer (5 μM)	1
BigDye™Terminator v1.1 Reaction Mix	1
BigDye™ Terminator v1.1Sequencing Buffer	1.5
Milli-Q H2O	5.5
Total Volume	10

Supplementary TABLE S19: Cycle Sequencing mixture formulation

Step	Temperature	Duration
1.	96°C	1 min
2.	96°C	10 s
3.	50°C	5 s
4.	60°C	4 min
Repeat Steps 2 to 4 for 30 times		
8.	4°C	-

Supplementary TABLE S20: PCR product purification

Purification of cycle sequencing products were conducted by pooled together with 45 μL SAM solution and 10 μL BigDye XTerminator™ bead solution in a MicroAmp™ Optical 96-Well Reaction Plate (Thermo Fisher Scientific). The plate was sealed using Microseal 'B' PCR Plate Sealing Film (Bio-rad) and was vortexed on a Digital Vortex-Genie™ 2 at 1,800 rpm for 30 minutes. The plate was then centrifuged at 1,000 x g for 2 minutes and ready to be sequenced.

Results. As shown in Table S21, among 10 clinical samples, 3 were identified positive, 7 were not identified by Sanger sequencing. 2 identified by Sanger sequencing were further confirmed as EML4 exon20-ALK exon20, and one was identified as EML4 exon 15-ALK exon 20.

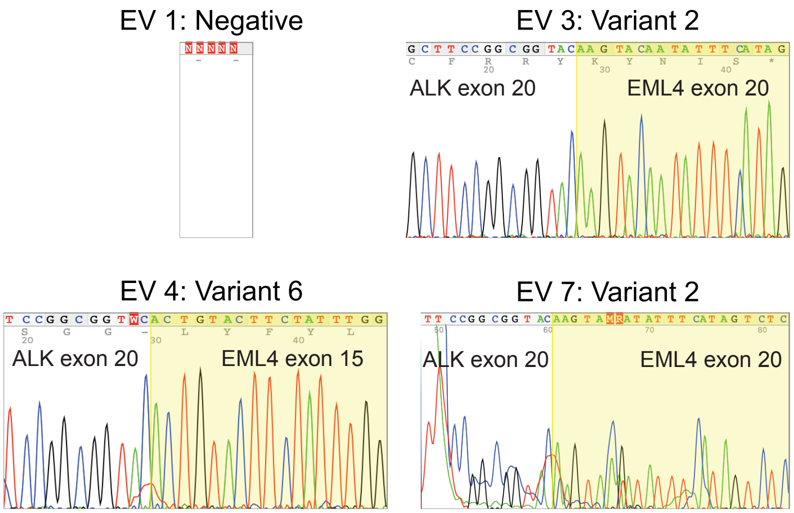

Supplementary FIG. S23: Example sanger traces of clinical EV samples.

Sample ID	Sanger Results	Sample ID	Sanger Results
EV 1	Negative	EV 6	Negative
EV 2	Negative	EV 7	EML4 exon 20 – ALK exon 20
EV 3	EML4 exon 20 – ALK exon 20	EV 8	Negative
EV 4	EML4 exon 15 – ALK exon 20	EV 9	Negative
EV 5	Negative	EV 10	Negative

Supplementary TABLE S21: Results of clinical NSCLC samples

[1] Conesa, A., Madrigal, P., Tarazona, S., Gomez-Cabrero, D., Cervera, A., McPherson, A., Szcześniak, M. W., Gaffney, D. J., Elo, L. L., Zhang, X., & Mortazavi, A. (2016). A survey of best practices for RNA-seq data analysis. Genome biology, 17, 13.

S9. Primer candidates dG° Calculation

Primer dG° calculation. The calculation of dG° of primer binding to the template, we used the parameter of dH and dS that was published by Santa Lucia [1]. The enthalpy and entropy can be found in Table S22, DNA bases 'ATCG' was represented by '1234' respectively. The dG° of each propagation sequence will change accordingly when the Temperature and Salinity change. We also included the dG° of each propagation sequence calculated at a Temperature(T) at 60° and a Salinity(S) of 0.18. The equation can be calculated as below:

$$\mathrm{dS}_s = \mathrm{dS(e.u.)} + 0.368 \cdot log(S) \tag{1}$$

When S = 0.18, the equation is

$$\mathrm{dS}_{0.18} = \mathrm{dS(e.u.)} + 0.368 \cdot log(0.18) \tag{2}$$

And when the temperatue is at $60°C$, the Gibbs free energy can be calculated as:

$$\mathrm{dG°}_{(60,0.18)} = \mathrm{dH} - (60 + 273.15) \cdot \mathrm{dS}_{0.18}/1000 \tag{3}$$

Example dG° calculation of one primer binding to the template is shown in Supplementary Fig. S24. Using the nearest neighbor parameters, the dG° of fP1 binding to the template at $60°C$ and a salinity of 0.18 can be calculated. Including a dG_{init} of 2.09 kcal/mol, the predicting dG° of fP1 is -11.89 kcal/mol and meet our requirements of primer dG° range from -10.5 kcal/mol to -12.5 kcal/mol.

Propagation Sequence	dH (kcal/mol)	dS (e.u.)	dG°($60°C$, 0.18) (kcal/mol)
11	-7.6	-21.3	-0.29
12	-7.2	-20.4	-0.19
13	-8.4	-22.4	-0.73
14	-7.8	-21	-0.59
21	-7.2	-21.3	0.11
22	-7.6	-21.3	-0.29
23	-8.2	-22.2	-0.59
24	-8.5	-22.7	-0.73
31	-8.5	-22.7	-0.73
32	-7.8	-21	-0.59
33	-8	-19.9	-1.16
34	-10.6	-27.2	-1.33
41	-8.2	-22.2	-0.59
42	-8.4	-22.4	-0.73
43	-9.8	-24.4	-1.46
44	-8	-19.9	-1.16

Supplementary TABLE S22: Nearest-Neighbor Parameters

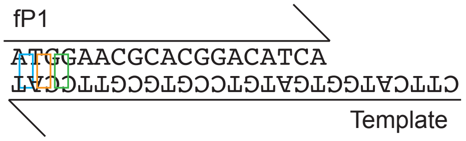

dG = dG(init) + dG(AT/TA) + dG(TG/AC) + dG(GA/CT)+ dG(AA/TT) + dG(AC/TG)
+ dG(CG/GC) + dG(GC/CG) + dG(CA/GT) + dG(AC/TG) + dG(CG/GC)
+ dG(GG/CC) + dG(GA/CT) + dG(AC/TG) + dG(CA/GT) + dG(AT/TA)
+ dG(TC/AG) + dG(CA/GT)

dG = dG(init) + dG(12) + dG(24) + dG (41) + dG(11) + dG(13)
+ dG(34) + dG(43) + dG(31) + dG(13) + dG(34)
+ dG(44) + dG(41) + dG(13) + dG(31) + dG(12)
+ dG(23) + dG(31)

dG = -11.89 kcal/mol

Supplementary FIG. S24: Example amplicon length variation from NGS reads.

---

[1] Santa Lucia J Jr, Hicks D. The thermodynamics of DNA structural motifs. Annu Rev Biophys Biomol Struct, 33:415-440. doi:10.1146/annurev.biophys.32.110601.141800 (2004).

S10. AmpliSeq Comparison experiments

Library preparation. A customized Ampliseq panel was ordered from illumina. Detailed AmpliSeq panel information including amplicon sequences, target coordinates are in supplementary excel file. Library preparation were performed following AmpliSeq's standard protocol, with only one modification to the first PCR cycles to be 23 rather than 20. A first try of a library preparation using 20-cycles amplification failed, after consulting illumina, we changed the first PCR cycles to be 23 to maximize the reaction performance.

PCR reaction mix was prepared by combining 2X AmpliSeq DNA Panel Pool (primer mix), Ampliseq HiFi mix, DNA template and Milli-Q H2O. DNA template was 10 ng sheared NA18562 per Panel Pool. The total reaction volume was 10μL in a 0.2mL Eppendorf PCR tube as shown in Table S23 and Table S24. Reaction mixture was placed on a Thermocycler C1000 (Bio-rad) followed the amplification protocol in Table S25. Following library preparation including FuPa clean up, ligation, Index amplification, and beads size selection were performed following AmpliSeq's standard protocol.

Final libraries were quantified by Qubit dsDNA HS Assay Kit (Thermo Fisher Scientific). Sizes of NGS libraries were measured using Bioanalyzer High Sensitivity DNA Assay (Agilent). DNA chip was run on the Agilent 2100 Bioanalyzer system. All the libraries were loaded on a Miseq Reagent V2 for obtaining pair-end reads on a Miseq sequencer(Illumina).

We prepared 4 libraries with different number of primer pools, input of template, with/ without size selection. Ideally the comparison of with/without the dimer digestion enzyme should be performed as well, however we were not sure the detailed composition of FuPa (which include at least three different enzymes) [1]. Therefore we only performed the libraries with FuPa clean-up. Detailed library information is in Table S26.

AmpliSeq was delivered in 2 primer panel pools originally, and we prepared the library using either 2 primer panel pools or 1 primer panel pool(mixed at 1:1 ratio from 2 primer panel pools), since that 1 primer panel pool is a more direct comparison to SADDLE (including 96 primer pairs in one pool).

Reagent	Add in Volume(μL)
5X AmpliSeq HiFi Mix	5
template (5ng /μL)	2.5
Milli-Q H2O	5
Total Volume	12.5

Supplementary TABLE S23: Ampliseq PCR mastermix formulation

Reagent	Add in Volume(μL)
Ampliseq PCR mastermix	5
2X AmpliSeq DNA Panel Pool 1/2	5
Total Volume	10

Supplementary TABLE S24: Ampliseq PCR reaction mixture formulation

Step	Temperature	Duration
1. Initial Denaturation	99°C	2 min
2. Denaturation	99°C	15 s
3. Annealing	60°C	4min
Repeat Steps 2 to 3 for 23 times		
Hold at 4°C		

Supplementary TABLE S25: AMP DNA amplification protocol

Library	AmpliSeq primer pool number	FuPa Digestion	Size Selection	Input DNA/primer pool
Lib1	2	Yes	Yes	10 ng genomic NA18562
Lib2	2	Yes	Yes	10 ng sheared NA18562
Lib3	1	Yes	Yes	10 ng sheared NA18562
Lib4	1	Yes	No	10 ng sheared NA18562

Supplementary TABLE S26: AmpliSeq library preparation information

Results.

AmpliSeq Bioanalzyer results. A shifted amplicon peak from around 140 bp before the FuPa clean up to 100 bp after the FuPa clean up can be observed (Supplementary Fig. S25). This result is accordance with our understanding that AmpliSeq's FuPa enzyme mixture can digest modified bases in primers [1]. Both primer dimer and primer sequence at the 5' end and 3' end of each amplicon can be cleaved and digested. Therefore, after FuPa clean up, targeted amplicon is shortened, and virtually no dimers can be observed from the Capillary electrophoresis figure.

Additional libraries were prepared by mixing primer panel 1 and primer panel 2 before the first multiplex PCR, therefore the single primer pool contained 100-plex primer pair, and it's more comparable to SADDLE PS3 (96 primer pairs). Gel figure of Library 3 (Final library) is shown in Supplementary Fig. S26

Library	Total reads	Bowtie2 aligned reads	On-target ratio	short reads(<60 bp)	short reads ratio(%)
Lib1	923005	604042	0.654	638	0.1
Lib2	3574440	2552771	0.714	8642	0.2
Lib3	2525251	1887350	0.747	22315	0.9
Lib4	1868016	1308782	0.701	118899	6.4

Supplementary TABLE S27: AmpliSeq Results

AmpliSeq NGS results.

AmpliSeq short reads ratio. In our data analysis pipline, we included a length of 60 bp filter to filter our short reads, and only align the rest longer reads to reference. The ratio of short reads in Library 1 and 2 are pretty low (<0.2%)(Table S27). Therefore even though identification of the exact number of dimers was not possible, we think that using AmpliSeq customized panel 100-plex, with standard protocol (2 primer panel pools, including FuPa clean up and size selection), the dimer ratio is low.

In Library 3 (single primer panel pool) and Library 4 (Single primer panel pool, without size selection), the short reads ratio are 0.9% and 6.4% respectively, suggesting the necessity to perform size selection during AmpliSeq's library preparation to get the most optimized results.

AmpliSeq on-target uniformity. We next had a look at the on-target reads of the libraries. Among the 96 loci, Ampliseq design failed on 1 locus, and 5 of them are covered by 2 primer pairs each, resulting a total of 100 primer pairs. The designed amplicon length was around 90 120 bp.

The sorted 100-plex on-target reads in Library 1 is shown in Supplementary Fig. S27(a). The majority of the on-target plex have a good uniformity, though less than 10 plex we observed much fewer reads.

A more direct comparison with SADDLE is to mix the AmpliSeq DNA Panel Pool 1 and Panel Pool 2 together as a single primer pool before the first multiplex PCR (Library 3). The on-target reads of each plex in Library 3 is shown in Supplementary Fig. S27(b).

Amplicon length variation. Amplicon length variation was observed using AmpliSeq's kit. For one target, the length of its amplicon after adapter trimming are usually more than one. As shown in Supplementary Fig. S28, the reads that amplifying regions of interest in gene APC have 4 different lengths. It is consistent to previous understanding that the FuPa enzyme mix can cleave primers at certain site, and the remaining sticky end can be further digested, leaving different length of amplicons. This is also the reason that we weren't able to perform dimer formation analysis as using SADDLE designed primer, given that: 1). primer sequences were not provided, and 2). primer digestion at both 5' end and 3' end of the amplicon prevented as to know the exact sequence at 5' end of each primer to run our dimer analysis pipline.

SADDLE Comparison. Comparison experiment was performed using SADDLE designed PS3 and other AmpliSeq's PCR reagents. PCR reaction mix was prepared by combining SADDLE multiplex primer mix, Ampliseq HiFi mix, DNA template and Milli-Q H2O (Table S28). The total reaction volume was 20μL in a 0.2mL

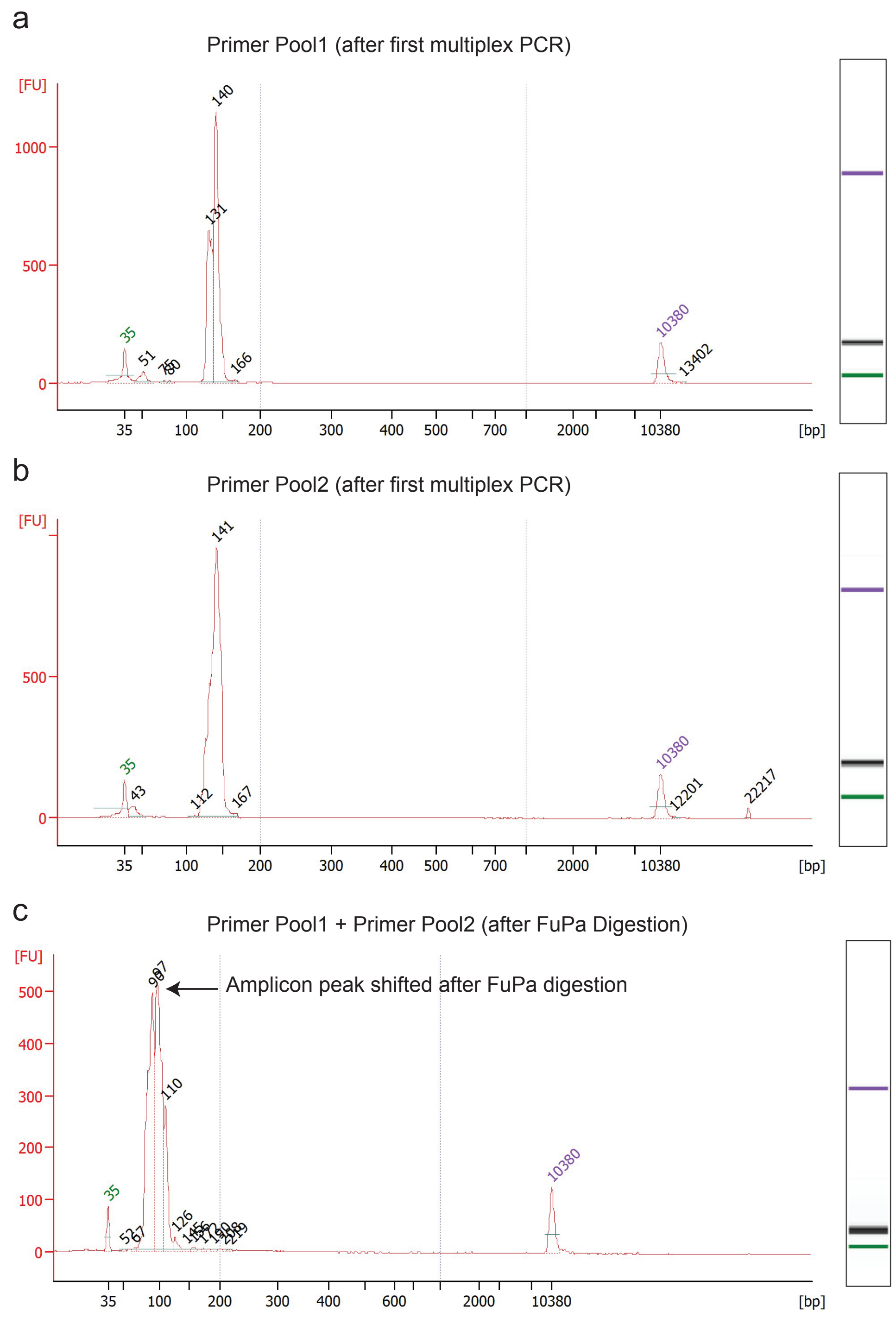

Supplementary FIG. S25: Capillary electrophoresis (Agilent Bioanalyzer 2100) analysis of products during library preparation. **(a)** First multiplex PCR product with AmpliSeq DNA Panel Pool 1, template input was 10 ng sheared NA18562. **(b)** First multiplex PCR product with AmpliSeq DNA Panel Pool 2, template input was 10 ng sheared NA18562. **(c)** PCR Product mixture (Panel Pool 1 and 2) after FuPa clean up. A shifted amplicon peak can be observed.

Eppendorf PCR tube. Final mastermix was placed on a Thermocycler C1000 (Bio-rad) followed the amplification protocol in Table S25.

As shown in Supplementary Fig. S29, we observed no amplicon length shift after FuPa digestion using SADDLE primer mix. SADDLE primers were purchased as standard desalted DNA oligonucleotides with no modifications (IDT), therefore would not be digested or cleaved by FuPa enzyme mixture. Nevertheless, even before the FuPa clean up, we observed little dimer using AmpliSeq HiFi mix, the dimer band from the gel shows even better performance than our previous experiment using Vent(exo-).

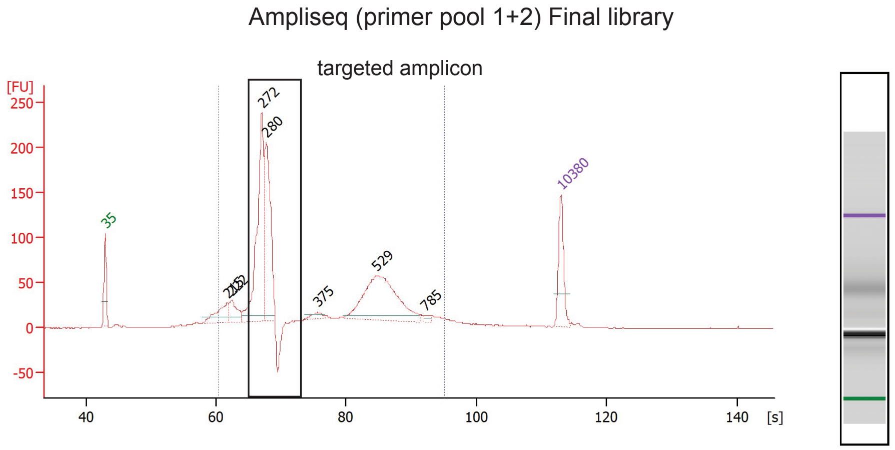

Supplementary FIG. S26: BioAnalyzer results of the final AmpliSeq library preparation. Peak of targeted amplicon is shown in black rectangular.

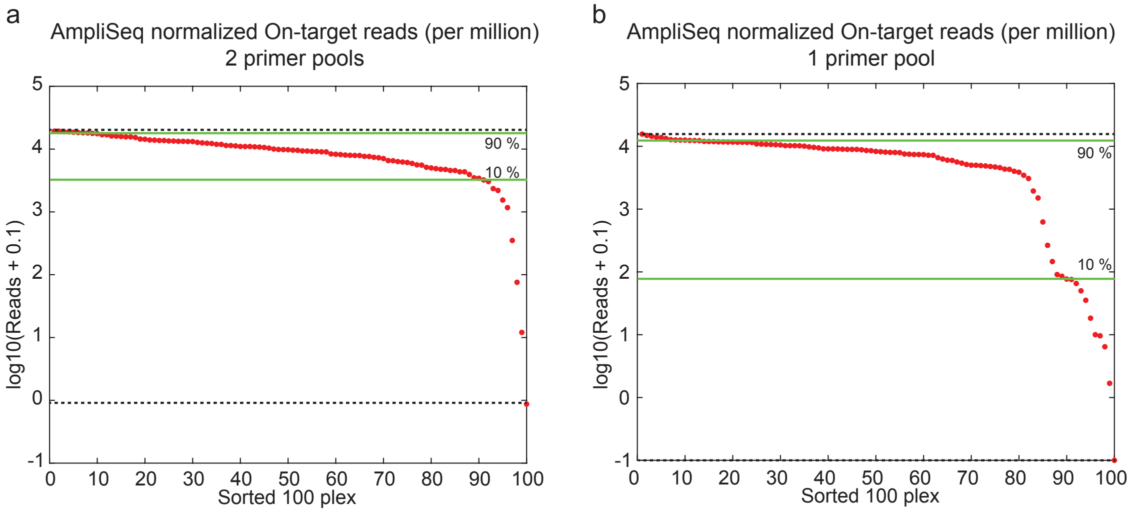

Supplementary FIG. S27: 100-plex on-target amplicons in AmpliSeq NGS library. **(a)** AmpliSeq 2 primer pools library. Here we plotted the distribution of reads normalized to 1 million reads. 90th percentile and 10th percentile of the on-target plex are plotted in green line. The maximum and minimum on-target reads are plotted in black dashed line. **(b)** AmpliSeq 1 primer pool library.

Reagent	Add in Volume(μL)
Ampliseq PCR mastermix	4
SADDLE primer mix	9
template (5ng /μL)	2
Milli-Q H2O	5
Total Volume	20

Supplementary TABLE S28: Ampliseq PCR reaction mixture formulation (SADDLE primer)

[1] John L. (2013). Methods and compositions for multiplex PCR. U.S. Patent Application No. 2013/0059762 (A1). Washington, DC: U.S. Patent and Trademark Office.

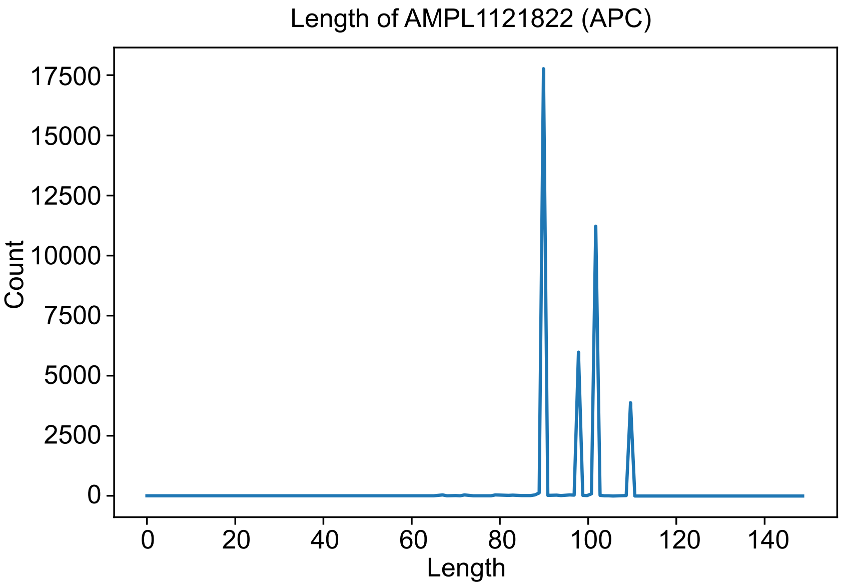

Supplementary FIG. S28: Example amplicon length variation from NGS reads.

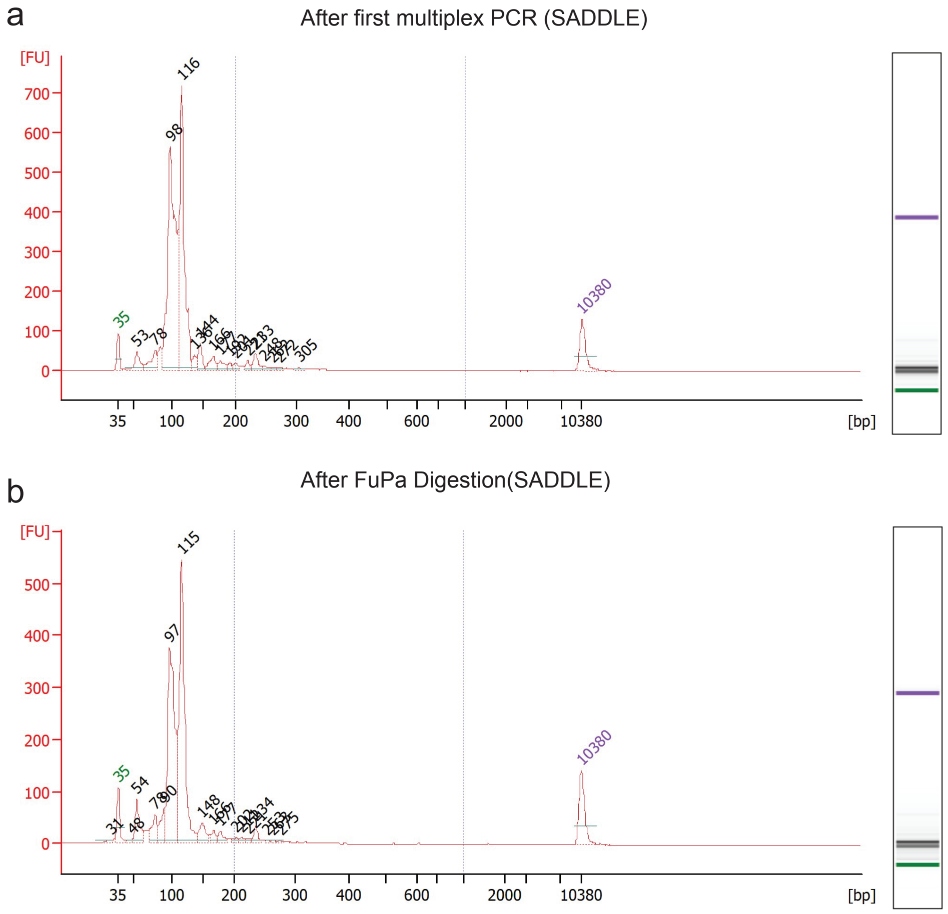

Supplementary FIG. S29: BioAnalyzer results during the library preparation. **(a)** First multiplex PCR product using SADDLE primer pool, template input was 10 ng sheared NA18562. **(b)** PCR Product using SADDLE primer pool after FuPa clean up. Amplicon length is almost identical as product before FuPa clean up. Note that during each step, column purification(Zymo) was performed without any size selection.

ALK Fusion Sequence

EML4 exon 13 -- ALK exon 20(EML4-ALK Variant 1)

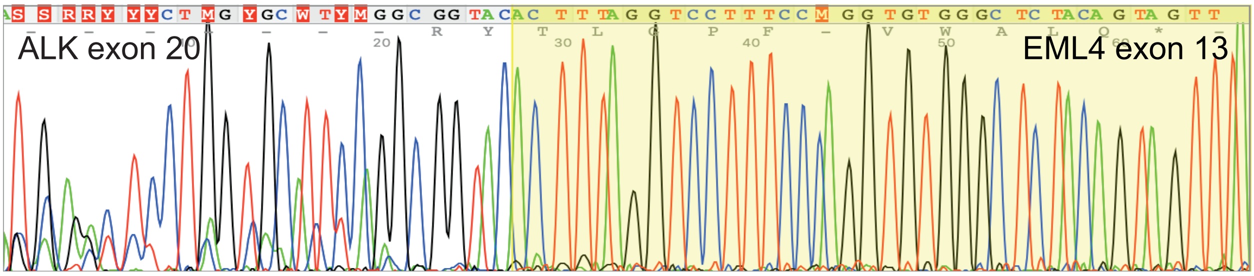

EML4 exon 20 -- ALK exon 20(EML4-ALK Variant 2)

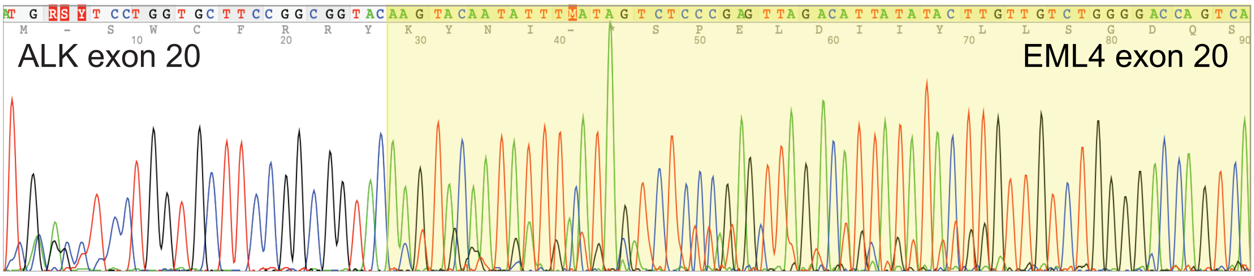

EML4 exon 6 -- ALK exon 20(EML4-ALK Variant 3a)

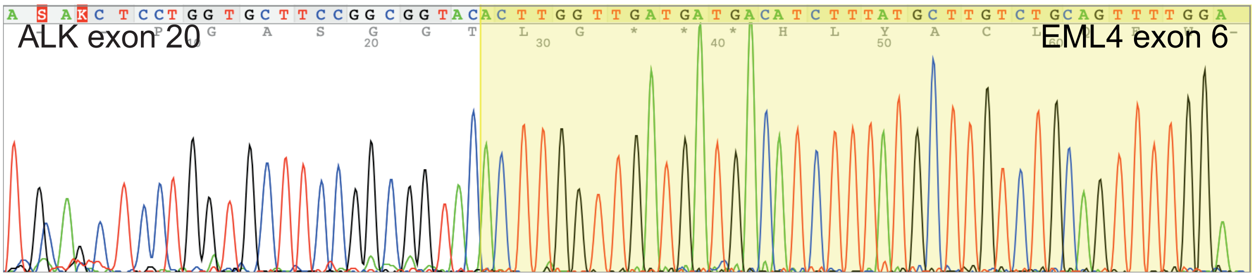

EML4 exon 6 -- ALK exon 20(EML4-ALK Variant 3b)

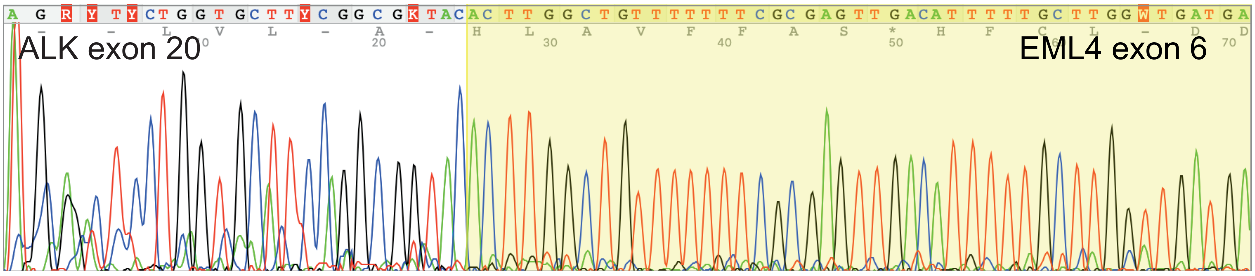

EML4 exon 6 -- ALK exon 20(EML4-ALK Variant 3c)

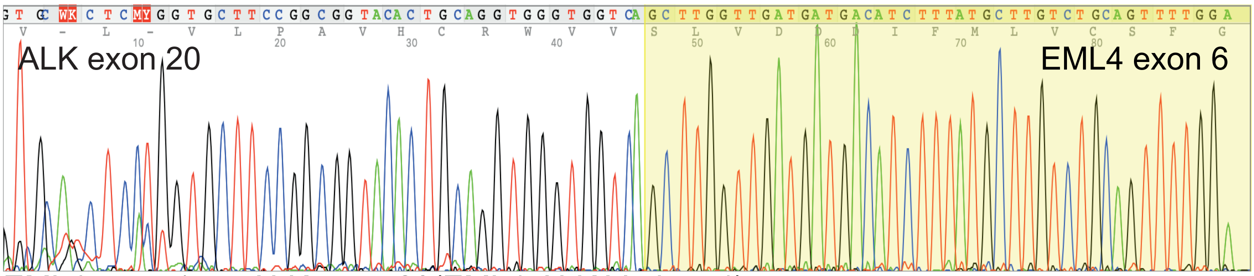

EML4 exon 15 -- ALK exon 20(EML4-ALK Variant 4)

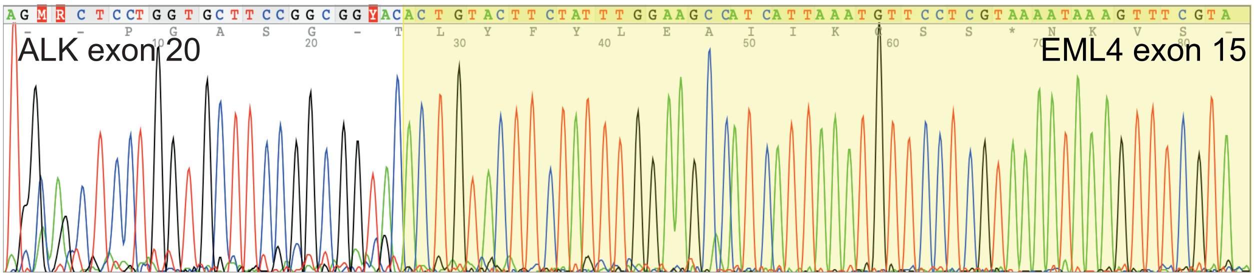

ALK Fusion Sequence

EML4 exon 18 -- ALK exon 20(EML4-ALK Variant 5)

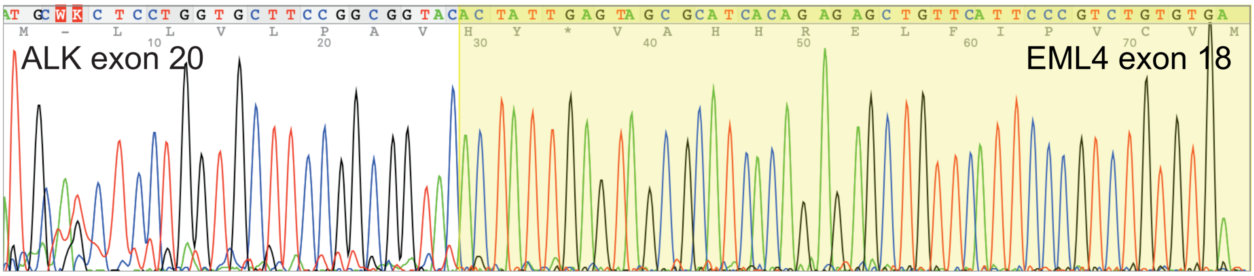

EML4 exon 2 -- ALK exon 20(EML4-ALK Variant 5a)

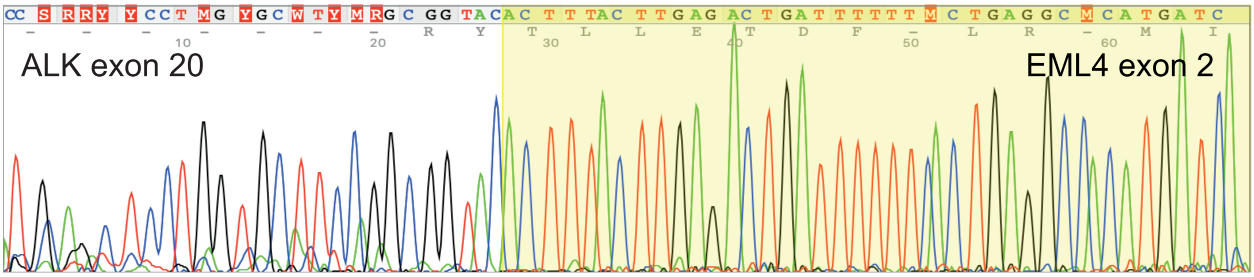

EML4 exon 14 -- ALK exon 20

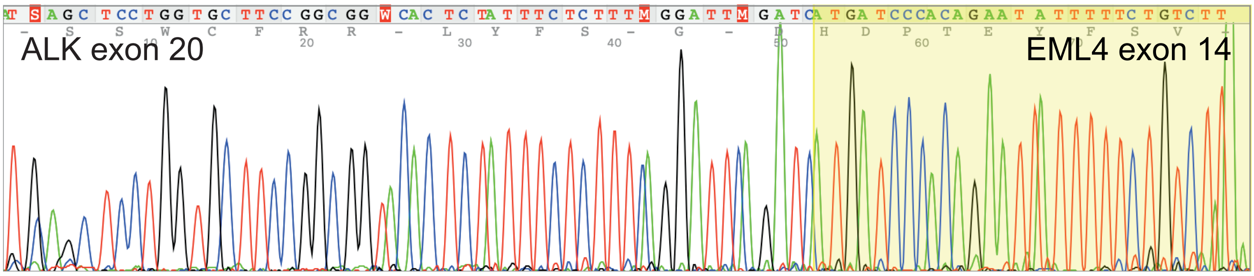

KIF5B exon 15 -- ALK exon 20

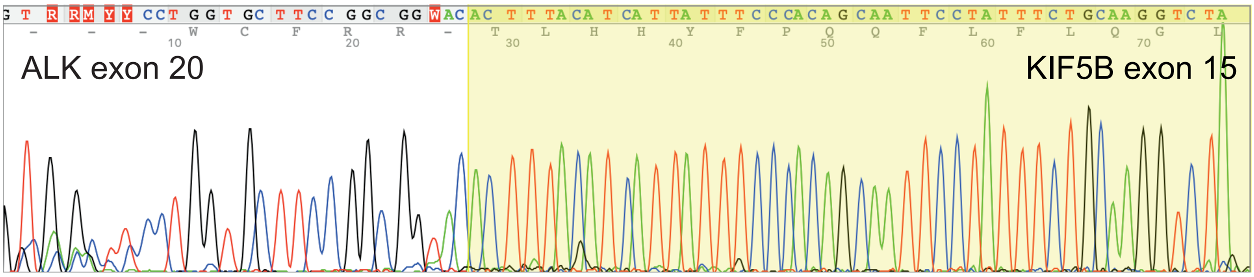

KIF5B exon 17 -- ALK exon 20

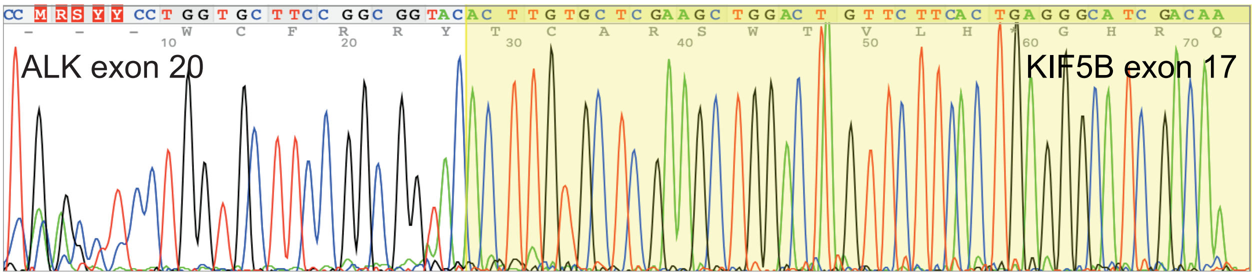

KIF5B exon 24 -- ALK exon 20

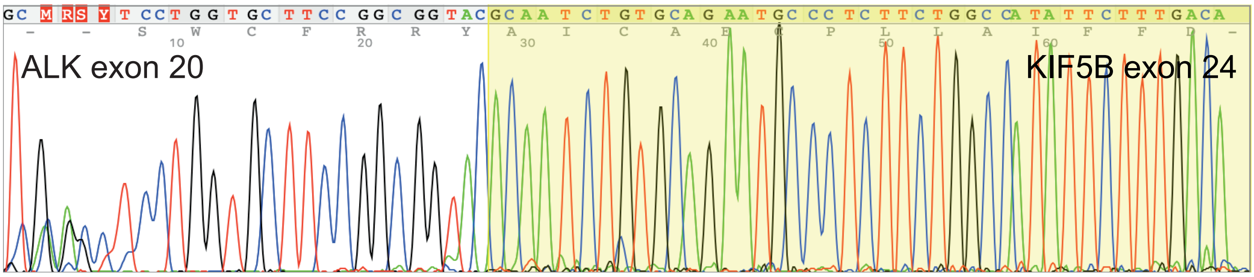

ALK Fusion Sequence

TFG exon 4 -- ALK exon 20

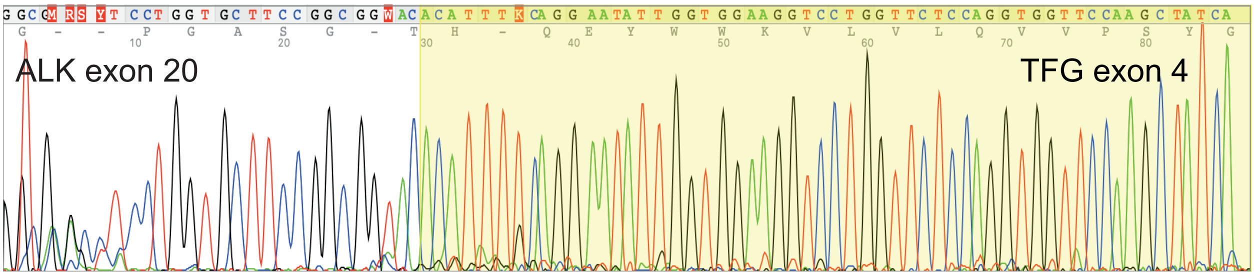

HIP1 exon 28 -- ALK exon 20

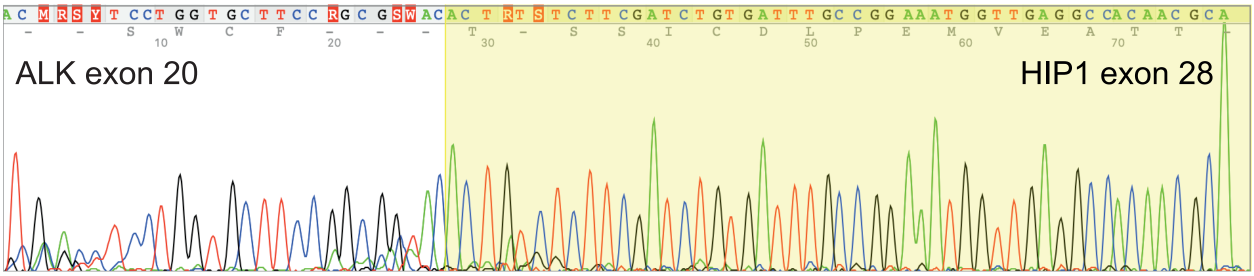

KLC1 exon 9 -- ALK exon 20

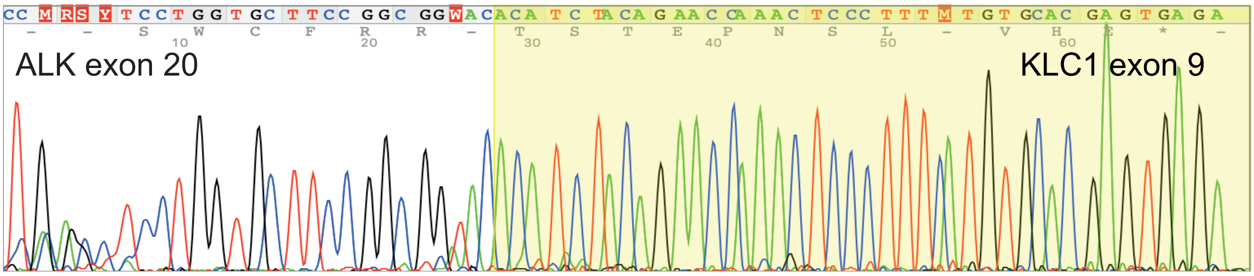

STRN exon 3 -- ALK exon 20

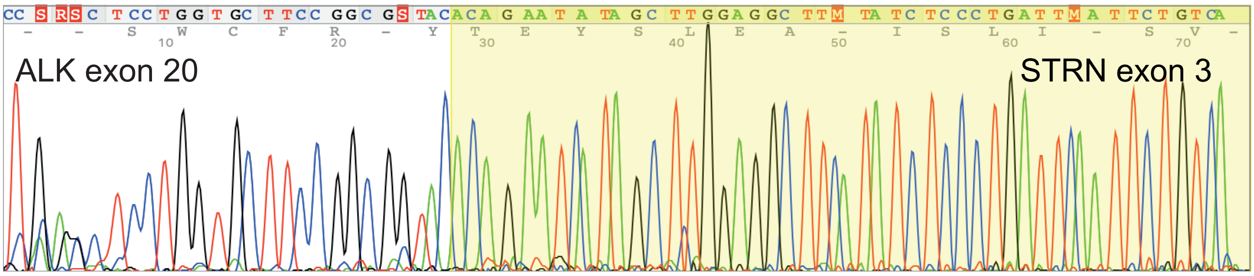

ATIC exon 7 -- ALK exon 20

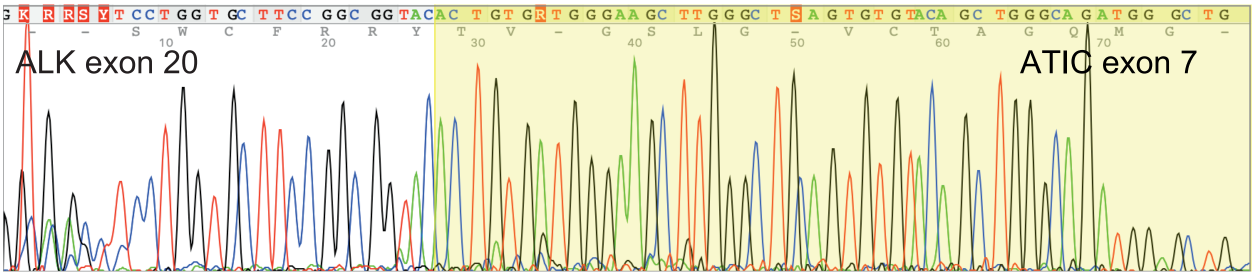

CARS exon 17 -- ALK exon 20

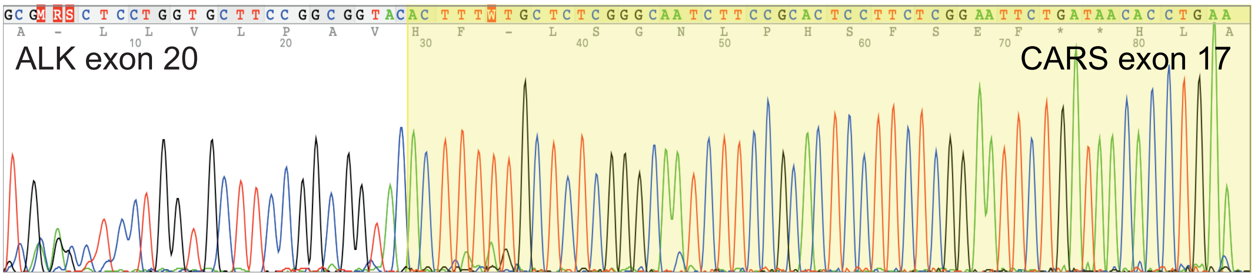

ALK Fusion Sequence

CLTC exon 31 -- ALK exon 20

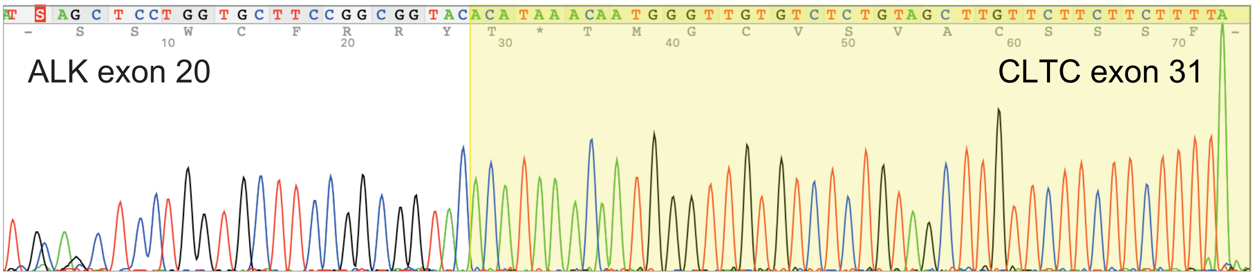

DCTN1 exon 26 -- ALK exon 20

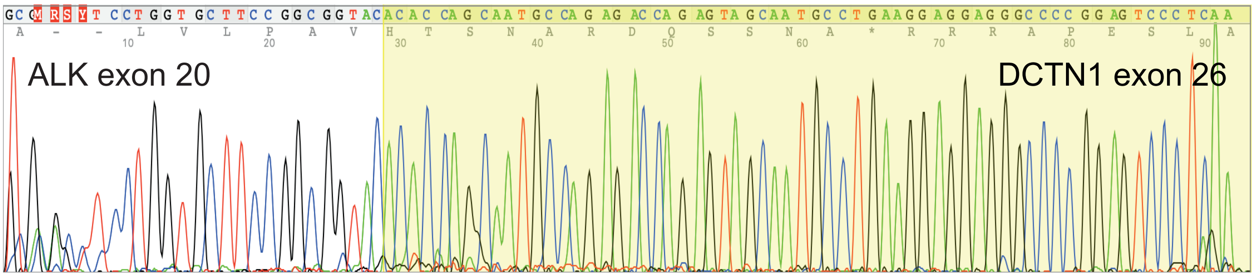

MSN exon 11 -- ALK exon 20

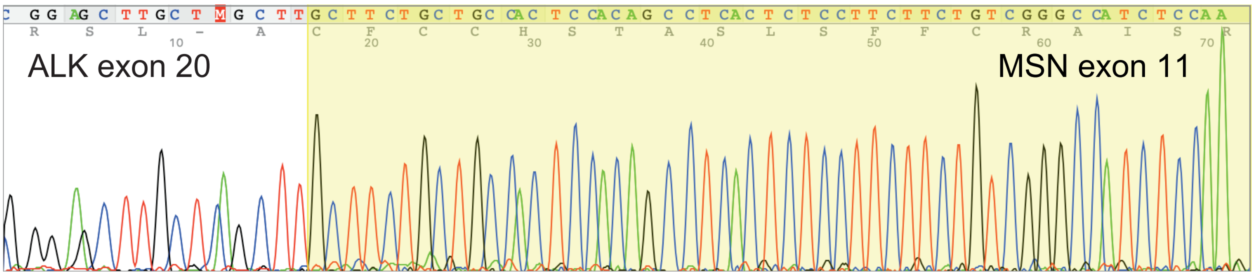

NPM1 exon 5 -- ALK exon 20

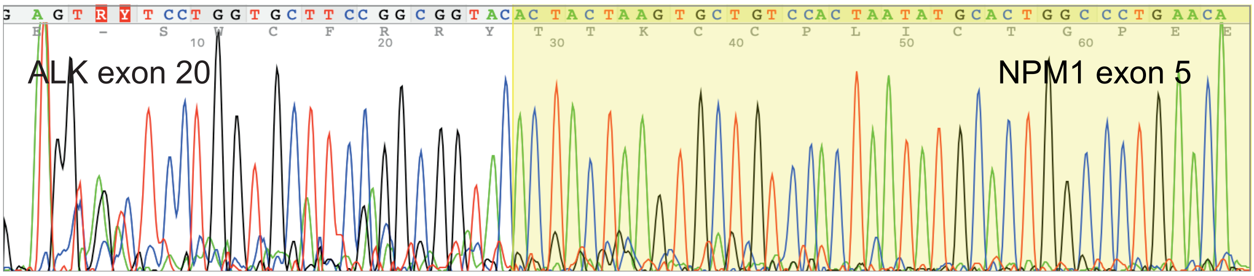

TPM3 exon 7 -- ALK exon 20

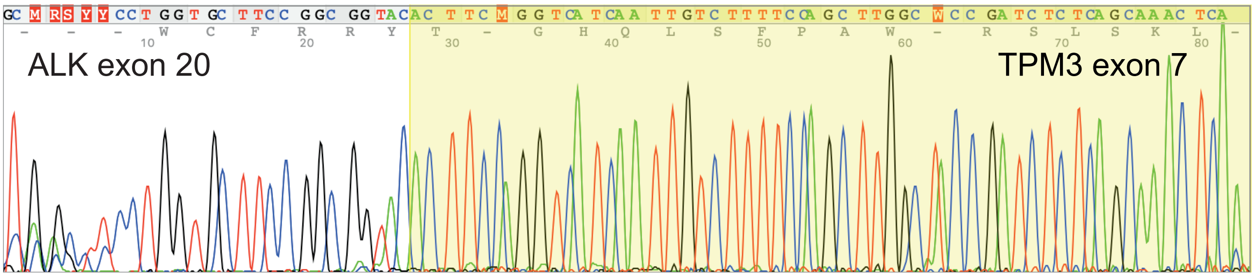

TPM4 exon 7 -- ALK exon 20

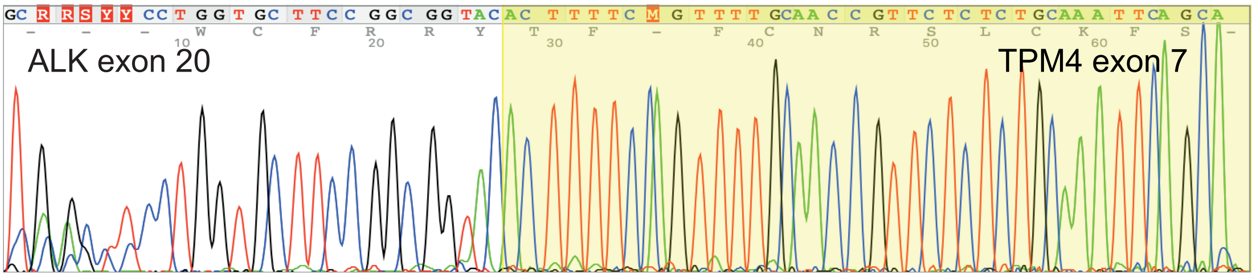

VCL exon 16 -- ALK exon 20

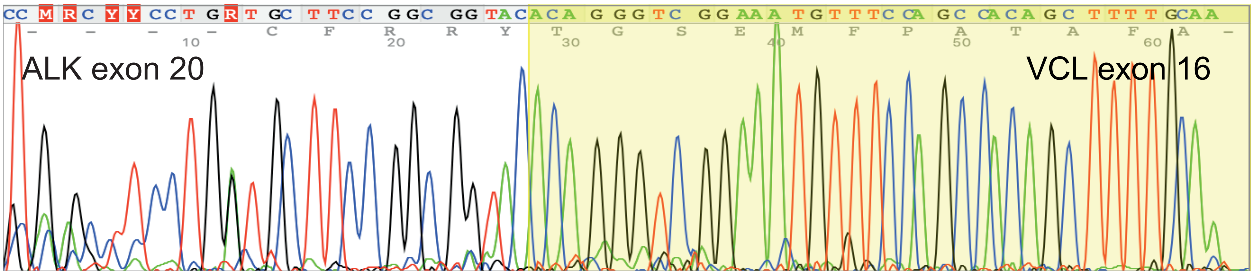

ROS1 Fusion Sequence

SDC4 exon 2 -- ROS1 exon 32

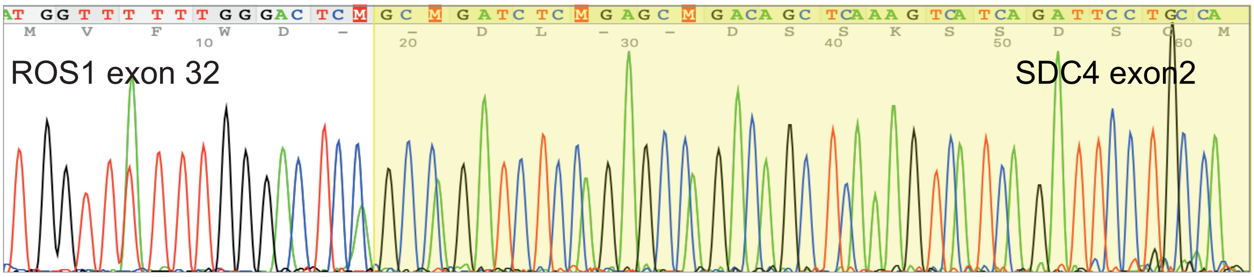

SDC4 exon 2 -- ROS1 exon 34

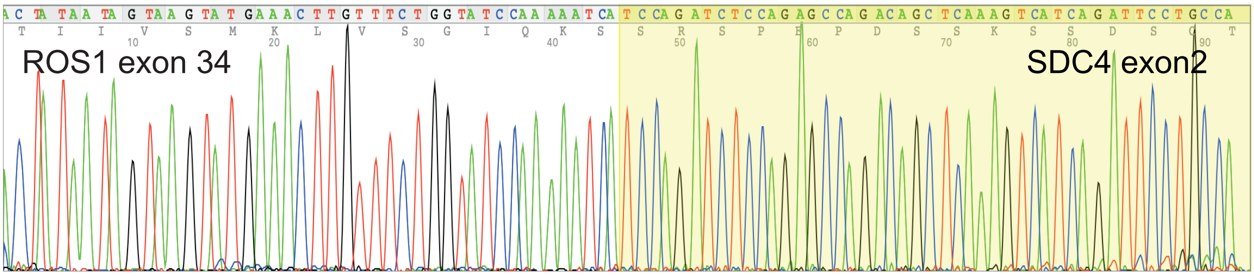

SDC4 exon 4 -- ROS1 exon 32

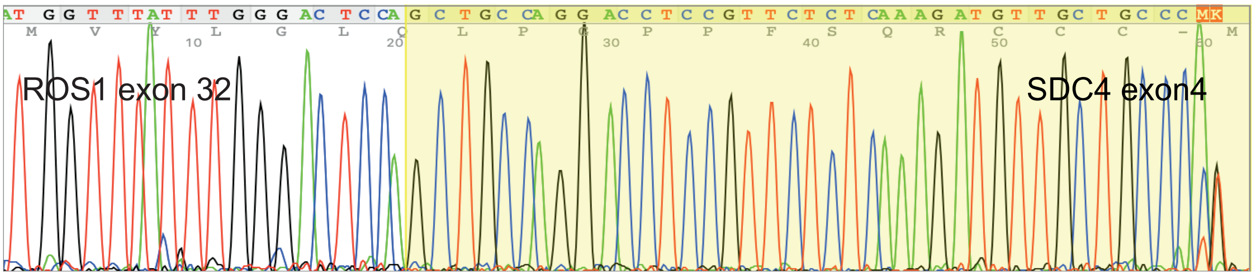

SDC4 exon 4 -- ROS1 exon 34

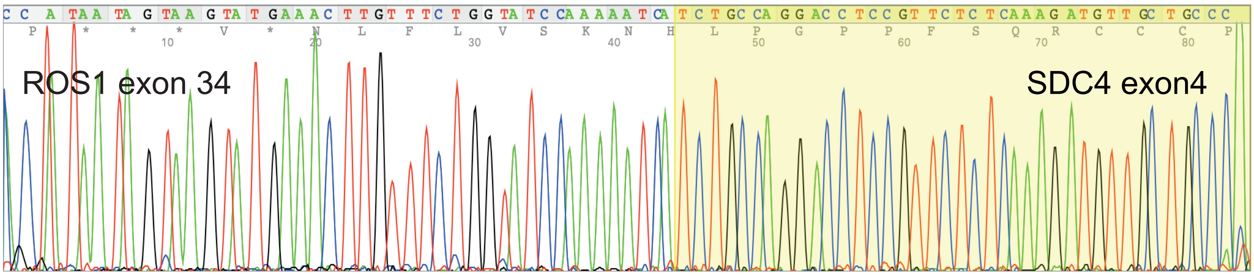

SLC34A2 exon 4 -- ROS1 exon 32

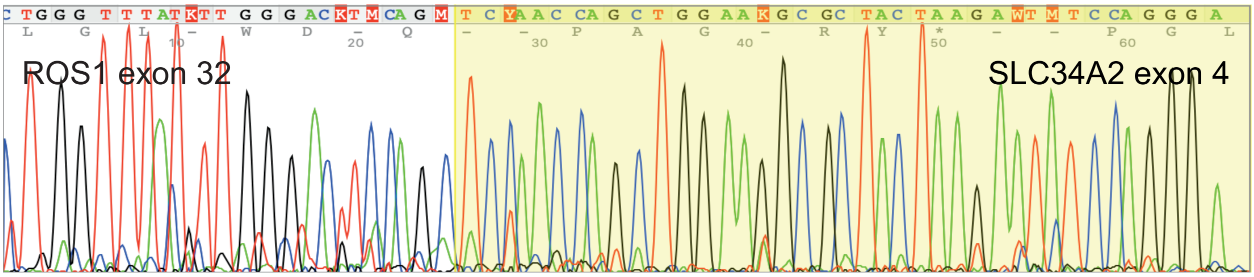

SLC34A2 exon 4 -- ROS1 exon 34

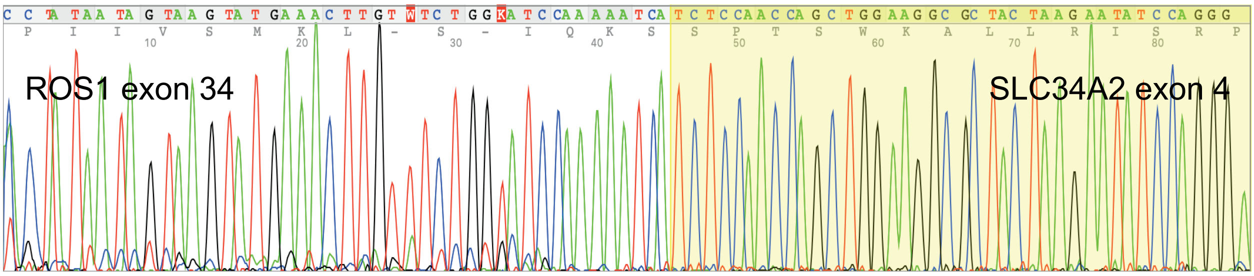

SLC34A2 exon 13 del -- ROS1 exon 32

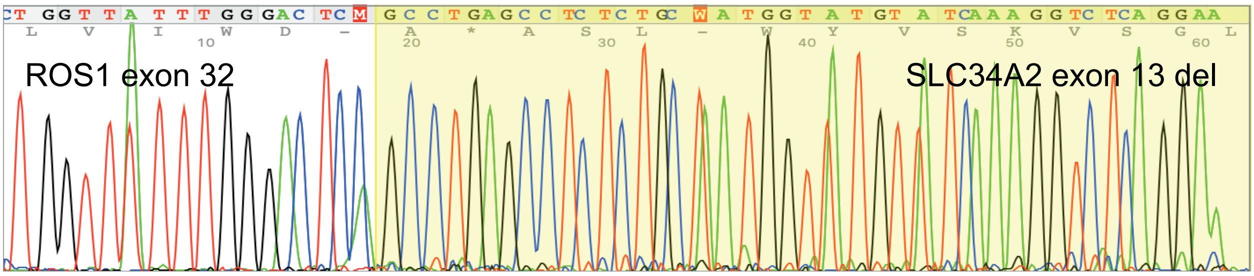

SLC34A2 exon 13 del -- ROS1 exon 34

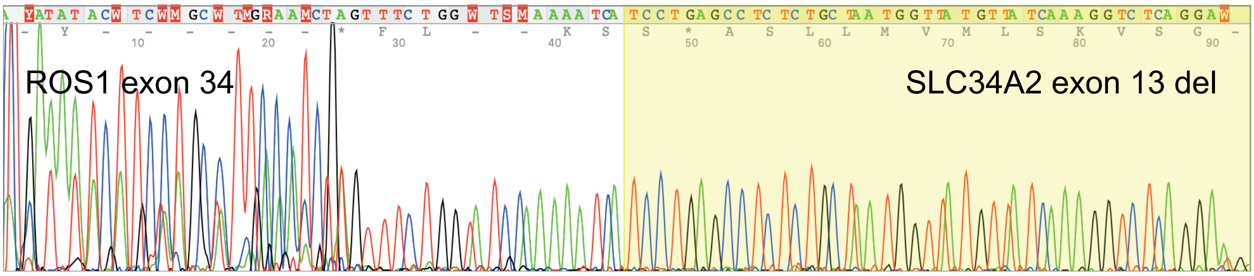

CD74 exon 6 -- ROS1 exon 32

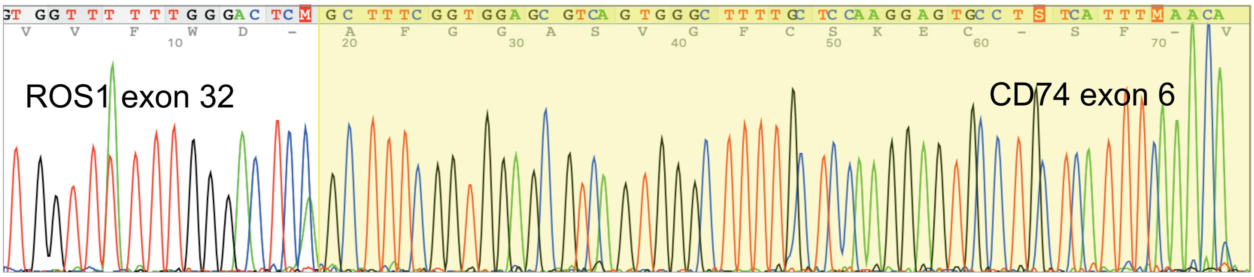

TPM3 exon 8 -- ROS1 exon 35

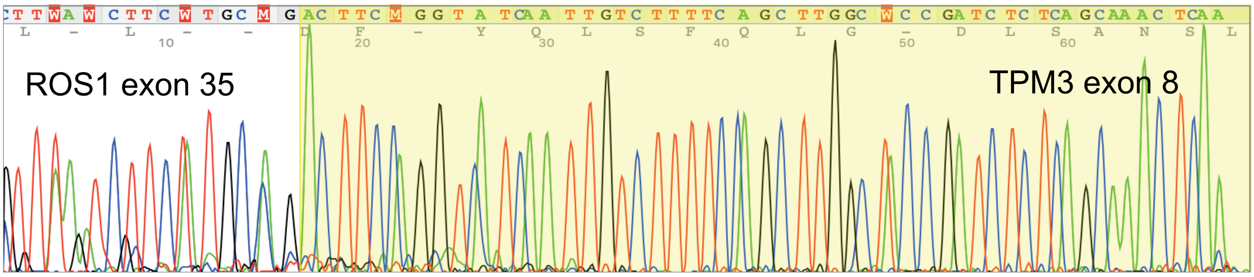

TPM3 exon 2 -- ROS1 exon 36

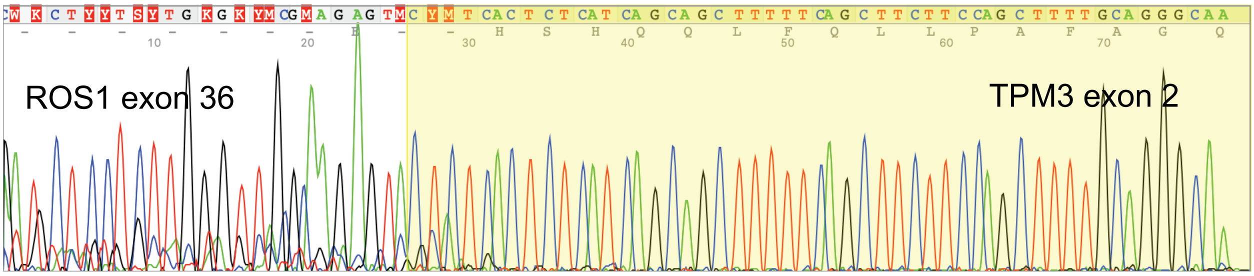

GOPC exon 7 -- ROS1 exon 35

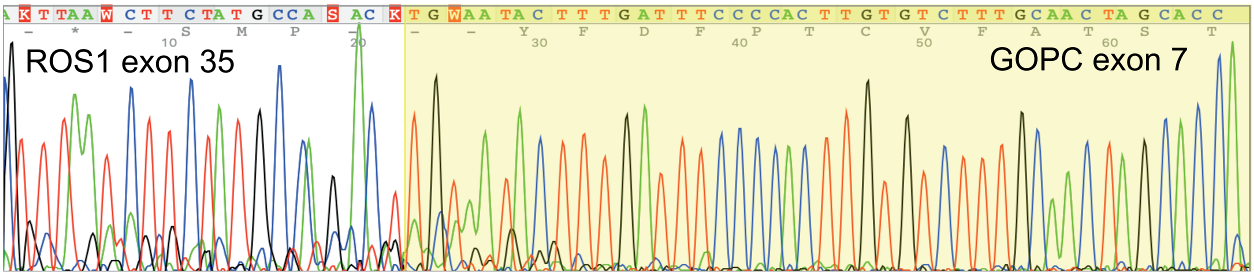

GOPC exon 4 -- ROS1 exon 36

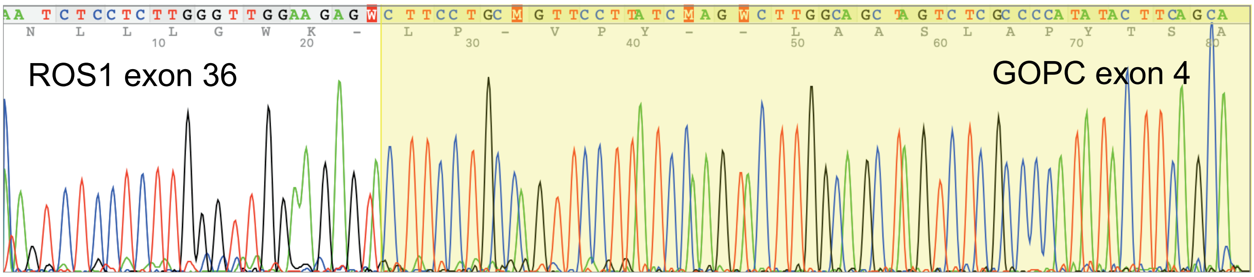

EZR exon 10 -- ROS1 exon 34

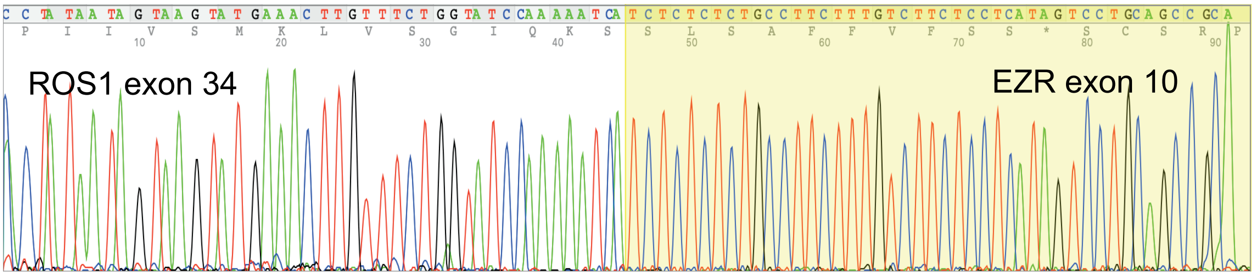

LRIG3 exon 16 -- ROS1 exon 35

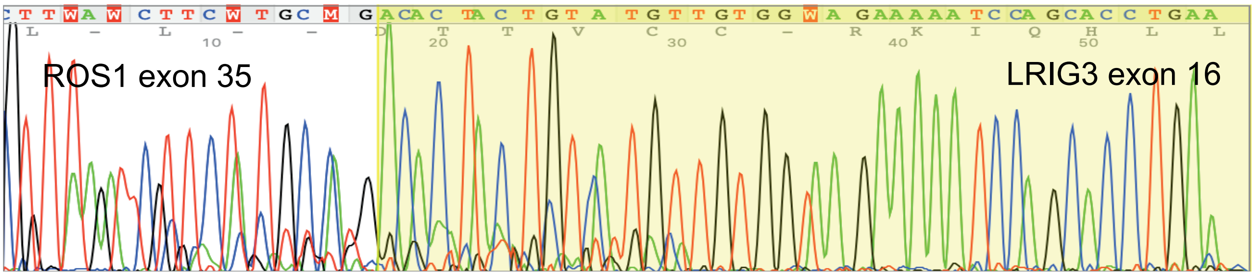

RET Fusion Sequence

KIF5B exon 15 -- RET exon 11

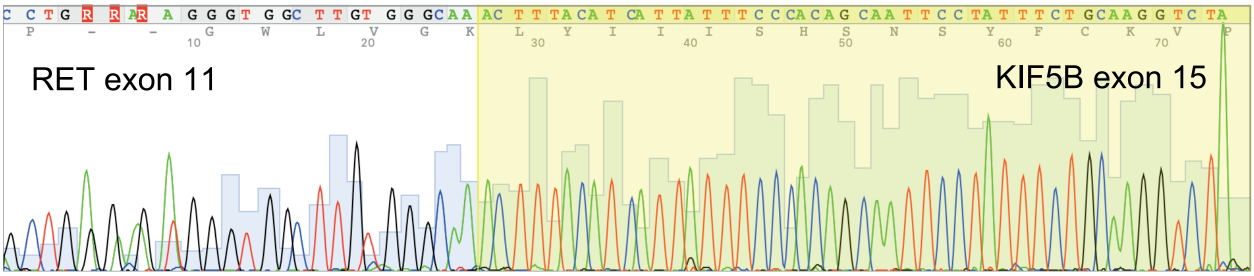

KIF5B exon 15 -- RET exon 12

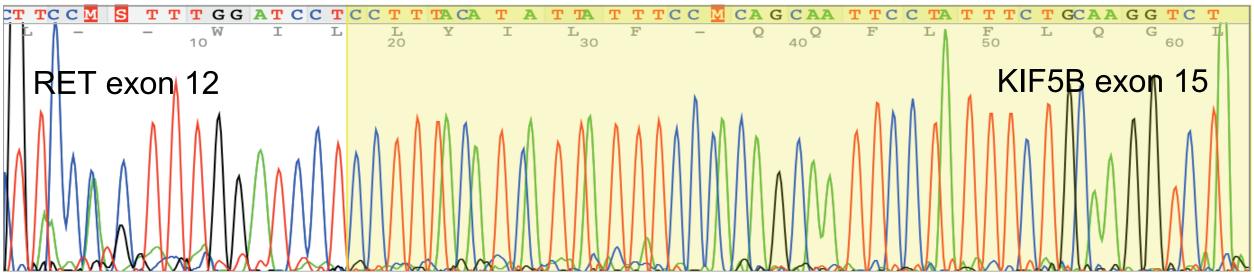

KIF5B exon 16 -- RET exon 12

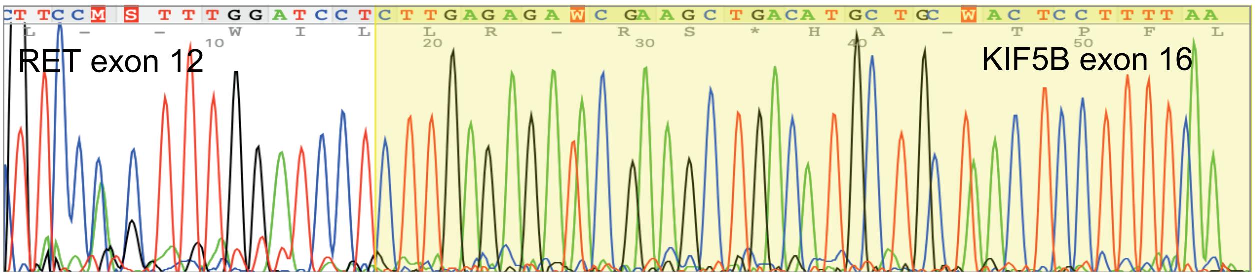

KIF5B exon 22 -- RET exon 12

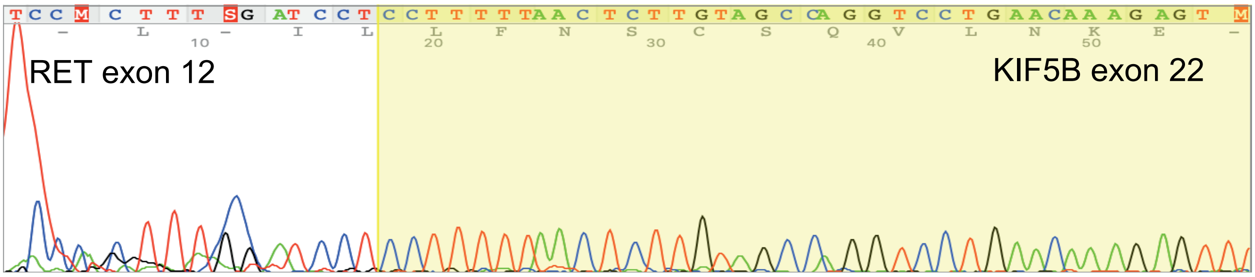

KIF5B exon 23 -- RET exon 12

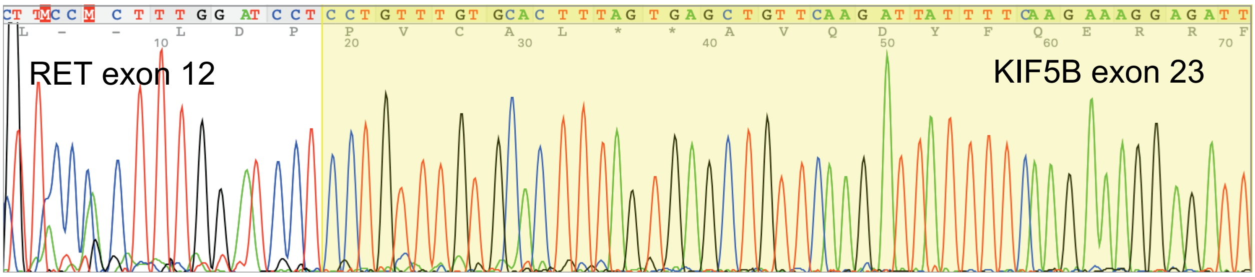

KIF5B exon 24 -- RET exon 8

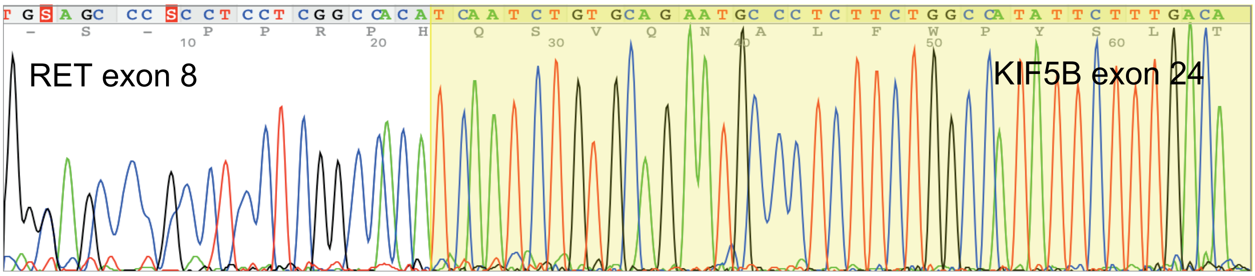

KIF5B exon 24 -- RET exon 11

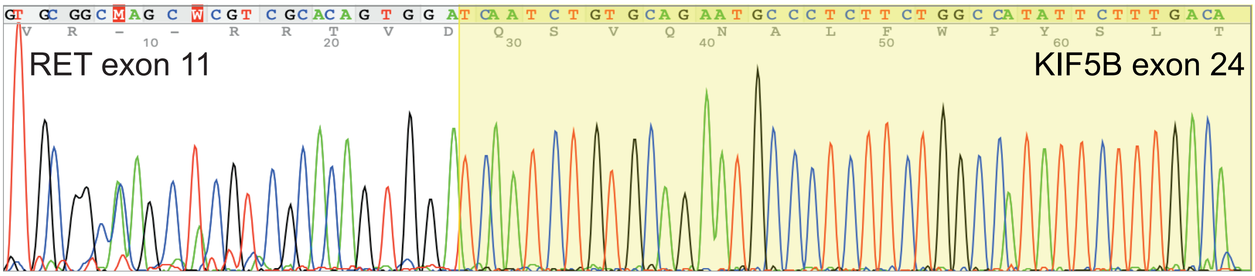

CCDC6 exon 1 -- RET exon 12

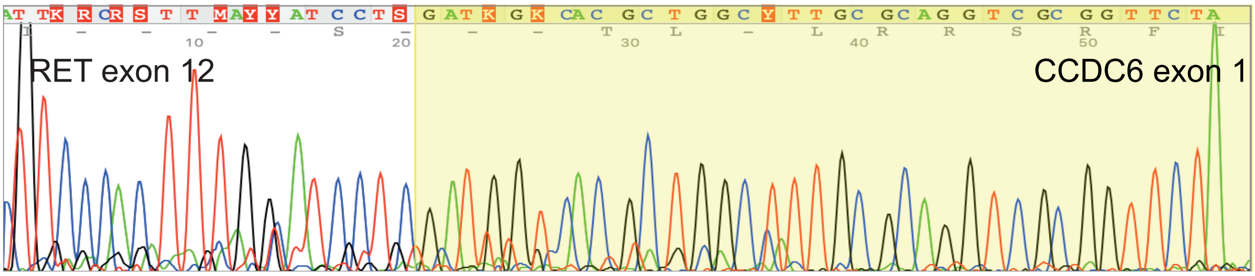

NCOA4 exon 6 -- RET exon 12

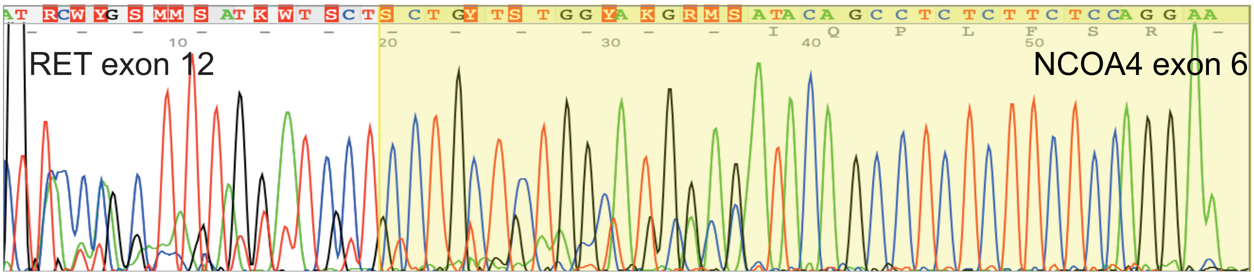

TRIM33 exon 16 -- RET exon 12

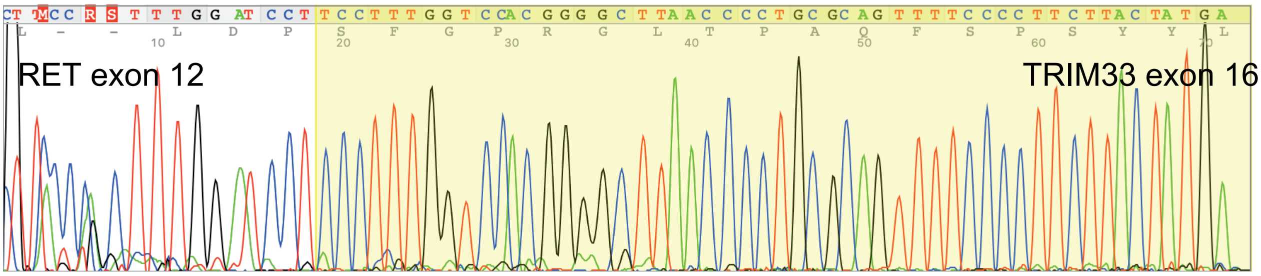

NTRK Fusion Sequence

TPM3 exon 7 -- NTRK1 exon 10

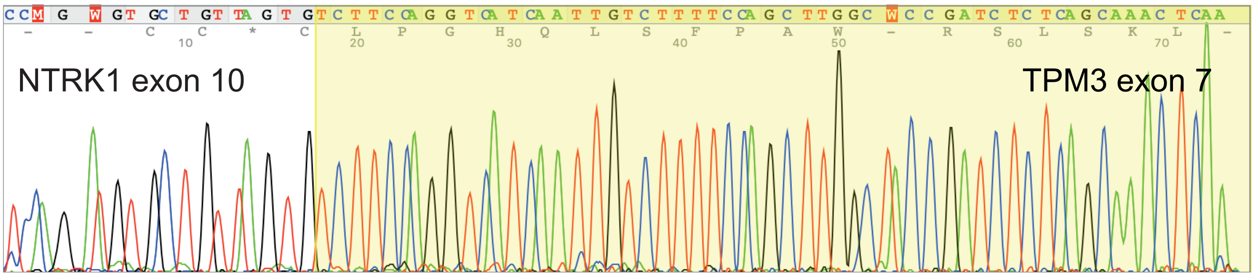

LMNA exon 2 -- NTRK1 exon 10

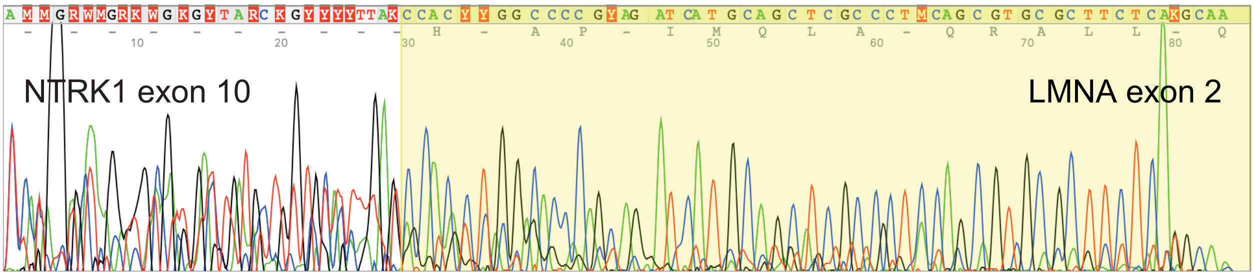

NACC2 exon 4 -- NTRK2 exon 13

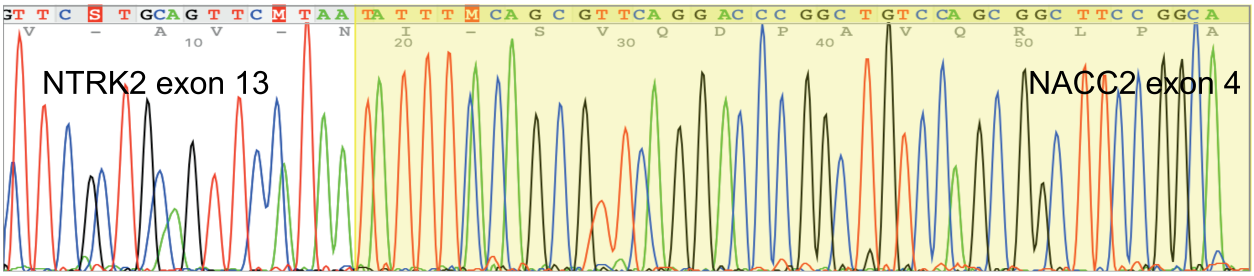

QKI exon 6 -- NTRK2 exon 16

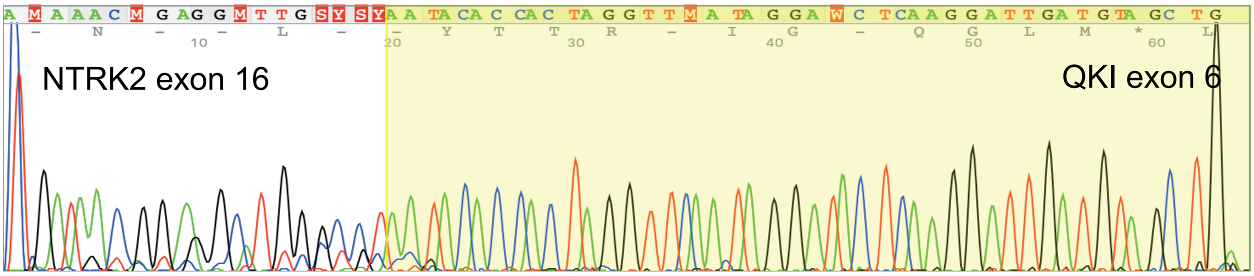

NTRK Fusion Sequence

ETV6 exon 4 -- NTRK3 exon 15

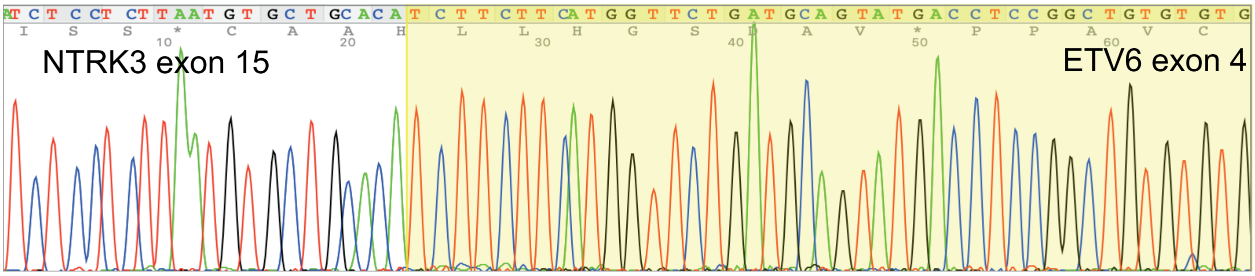

ETV6 exon 5 -- NTRK3 exon 15

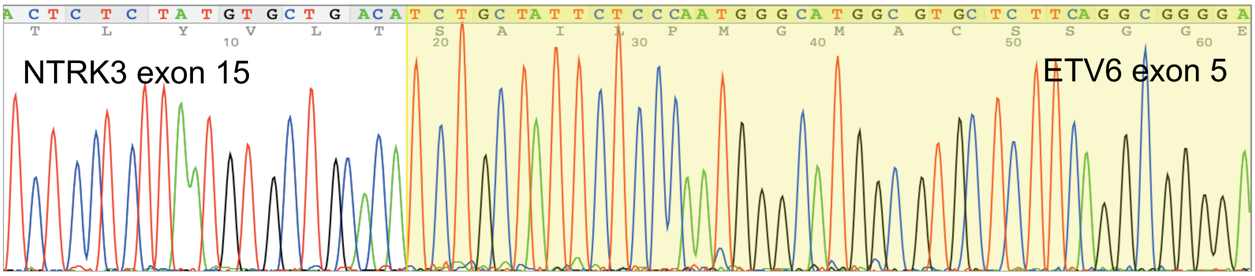

Supplement: Supplementary file 1 — Supplementary Information [file 41467_2022_29500_MOESM1_ESM.pdf]
