# Supplementary material for: Designing highly multiplex PCR primer sets with Simulated Annealing Design using Dimer Likelihood Estimation (SADDLE)
Source: Nat Commun. 2022 Apr 11;13:1881. doi: 10.1038/s41467-022-29500-4 (PMC9001684; doi:10.1038/s41467-022-29500-4)
Supplement: Supplementary file 4 — Description of Additional Supplementary Files [file 41467_2022_29500_MOESM4_ESM.pdf]

Title: Supplementary Data 1.
Description: Primer sequences for all experiments.

Title: Supplementary Data 2.
Description: Badness of all primer pairs of PS1, PS2 and PS3.

Title: Supplementary Data 3.
Description: Number of dimer reads of all primer pairs of PS1, PS2 and PS3.
